# Genetic Differentiation and Migration Fluxes of Viruses from Melon Crops and Crop Edge Weeds

**DOI:** 10.1128/jvi.00421-22

**Published:** 2022-08-04

**Authors:** Ayoub Maachi, Livia Donaire, Yolanda Hernando, Miguel A. Aranda

**Affiliations:** a Abiopep S.L., Parque Científico de Murcia, Complejo de Espinardo, Espinardo, Murcia, Spain; b Centro de Edafología y Biología Aplicada del Segura (CEBAS)-CSIC, Department of Stress Biology and Plant Pathology, Espinardo, Murcia, Spain; University of Maryland, College Park

**Keywords:** high-throughput sequencing, CCYV, CmEV, CABYV, ToLCNDV, virus reservoirs, migration fluxes, genetic differentiation

## Abstract

Weeds surrounding crops may act as alternative hosts, playing important epidemiological roles as virus reservoirs and impacting virus evolution. We used high-throughput sequencing to identify viruses in Spanish melon crops and plants belonging to three pluriannual weed species, *Ecballium elaterium*, *Malva sylvestris*, and *Solanum nigrum*, sampled at the edges of the crops. Melon and *E. elaterium*, both belonging to the family *Cucurbitaceae*, shared three virus species, whereas there was no virus species overlap between melon and the other two weeds. The diversity of cucurbit aphid-borne yellows virus (CABYV) and tomato leaf curl New Delhi virus (ToLCNDV), both in melon and *E. elaterium*, was further studied by amplicon sequencing. Phylogenetic and population genetics analyses showed that the CABYV population was structured by the host, identifying three sites in the CABYV RNA-dependent RNA polymerase under positive selection, perhaps reflecting host adaptation. The ToLCNDV population was much less diverse than the CABYV one, likely as a consequence of the relatively recent introduction of ToLCNDV in Spain. In spite of its low diversity, we identified geographical but no host differentiation for ToLCNDV. Potential virus migration fluxes between *E. elaterium* and melon plants were also analyzed. For CABYV, no evidence of migration between the populations of the two hosts was found, whereas important fluxes were identified between geographically distant subpopulations for each host. For ToLCNDV, in contrast, evidence of migration from melon to *E. elaterium* was found, but not the other way around.

**IMPORTANCE** It has been reported that about half of the emerging diseases affecting plants are caused by viruses. Alternative hosts often play critical roles in virus emergence as virus reservoirs, bridging host species that are otherwise unconnected and/or favoring virus diversification. In spite of this, the viromes of potential alternative hosts remain largely unexplored. In the case of crops, pluriannual weeds at the crop edges may play these roles. Here, we took advantage of the power of high-throughput sequencing to characterize the viromes of three weed species frequently found at the edges of melon crops. We identified three viruses shared by melon and the cucurbit weed, with two of them being epidemiologically relevant for melon crops. Further genetic analyses showed that these two viruses had contrasting patterns of diversification and migration, providing an interesting example on the role that weeds may play in the ecology and evolution of viruses affecting crops.

## INTRODUCTION

Emerging high-throughput sequencing (HTS) technologies are of relatively recent use in phyto-virology and are very powerful, allowing for the characterization of plant viromes, the detection of known viruses, and the discovery of novel ones (1). These technologies have been successfully used with several crop species (2–5). Weeds surrounding crops may act as alternative hosts, playing important epidemiological roles as virus reservoirs and impacting virus evolution. HTS could help detect the potential of weeds to act as reservoirs and alternative hosts for viruses infecting crops, by the comparison of viromes in a fast and efficient manner. For instance, a study comparing the diversity of viral populations between tomato plants and neighboring *Solanum nigrum* plants using HTS showed a large variability in virome richness, but with little overlap of the viruses found in both species (6). In another study, authors examined the influence of four different habitats on the evolution of watermelon mosaic virus (WMV), reporting that the WMV genetic diversity was structured by habitat types and host species (7). Also, Juárez et al. (8) showed that the tomato leaf curl New Delhi virus (ToLCNDV) population infecting Datura stramonium in Spain displayed higher levels of within-host genetic diversity when compared to crops (8). There are other studies using metagenomics for virus detection and diversity characterization in weeds and wild plants, but to our knowledge, none of them have analyzed the migration fluxes of viruses infecting weeds and crops (6, 8–11).

Melon (*Cucumis melo* L., family *Cucurbitaceae*) is an important crop cultivated for fresh fruit consumption. The total world production has been estimated at 28.5 million tons in 2020 (www.fao.org), with Spain being one of the main melon producers in the world. Viral infections seriously affect the quality and the yield of agricultural products, and epidemics of emerging viruses represent increasing and significant threats for sustainable crop production. There are more than 35 different viruses reported affecting cucurbits (12), and some of them have been identified in Spanish melon crops causing epidemics with serious economic repercussions (13, 14). Among them, cucurbit aphid-borne yellows virus (CABYV), ToLCNDV, and WMV appear to be the viruses prevalent in Spain (8, 13, 15). CABYV (genus *Polerovirus*, family *Solemoviridae*) can infect melon, cucumber, squash, and watermelon plants, where it remains confined to the phloem causing yellowing symptoms (16, 17). CABYV has a (+) single-stranded (ss) RNA genome and is transmitted in a circulative nonpropagative manner by at least two aphid species, Aphis gossypii and Myzus persicae (17). The genetic diversity of CABYV Spanish populations is large (18), suggesting multiple introductions and/or rapid diversification. ToLCNDV is a bipartite begomovirus with two circular ssDNA genome components (DNA-A and DNA-B) (19, 20). ToLCNDV is limited to the plant phloem and is transmitted in a circulative persistent manner by the whitefly Bemisia tabaci (20, 21). This virus has a wide host range with 43 different plant species identified so far, mainly belonging to the families *Solanaceae* and *Cucurbitaceae* (22–25). The Mediterranean population of ToLCNDV is genetically very homogeneous with no clustering patterns identified thus far (8). WMV, with a (+) ssRNA genome, belongs to the genus *Potyvirus* and is distributed worldwide. It is transmitted in a nonpersistent manner by aphids and has a broad host range of more than 100 plant species belonging to 27 families, including many weeds that can host the virus between crop seasons (14, 26).

Here, we used an HTS approach to identify the viruses present in melon crops in the Murcia, Madrid, and Castilla-La Mancha regions of Spain. Our data corroborated results of previous surveys in the area, but also identified cucumis melo endornavirus (CmEV), cucurbit chlorotic yellows virus (CCYV), and tobacco mild green mosaic virus (TMGMV) for the first time in melon crops in this country. We also assessed the potential of three important weed species as virus reservoirs for melon infection; one of the species, *Ecballium elaterium*, showed a significant virome overlap with melon, including CABYV, ToLCNDV, and TMGMV. An *E. elaterium*-specific lineage was identified for CABYV; interestingly, selection seemed to act differentially over CABYV in the crop and the weed hosts. Finally, we studied the population structure of CABYV and ToLCNDV both in melon and *E. elaterium*, testing possible genetic differentiation and migration hypotheses between the host viral subpopulations.

## RESULTS

### HTS of melon and associated weeds.

**FIG 1:**
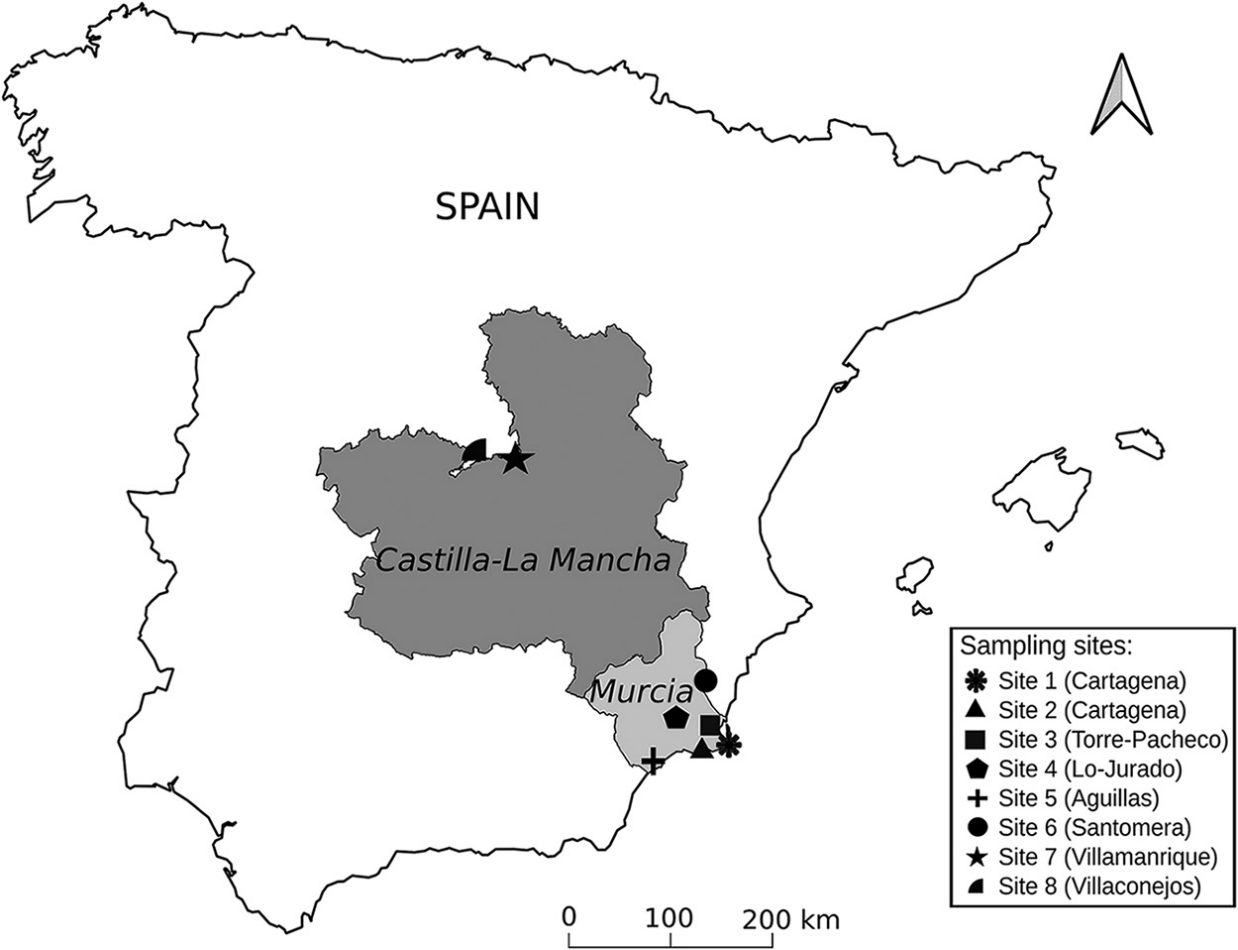
Map showing the sampling regions and the location of the melon fields. The sampling regions (Murcia and Castilla-La Mancha, Spain) are highlighted in gray. The sites, from where the samples of melon and associated weeds were collected, are represented using the different symbols shown in the legend.

We sampled melon plants and weeds belonging to three species, *E. elaterium*, *S. nigrum*, and *Malva sylvestris*, from eight different sites in the Murcia, Madrid, and Castilla-La Mancha regions of Spain (Fig. 1). These regions have a long tradition of melon cultivation and contribute the most to the Spanish melon production (MAPA, 2020). The melon plants exhibited yellowing, leaf curling, and/or mosaics (Fig. S1A to C), whereas the weed plants, which grew spontaneously on the edges of the fields (Fig. S1D and F), were almost all asymptomatic except for a few exceptions that showed general or partial chlorosis or mosaics (Fig. S1E and F). In total, we included 218 samples in the analysis (Table 1). Total RNA was extracted from pools per species from each site, and then aliquots of the RNA preparations from different sites were mixed for each species to construct four libraries, one per plant species. After sequencing using the Illumina technology, we obtained 95 to 97 M reads per library, which were processed following a bioinformatics pipeline that we previously developed for virus detection (27). Briefly, raw reads were cleaned of adapter sequences and filtered by quality and assembled into long contigs (Table S1), which were used as the query to search for virus sequences against the Refseq virus database. We then remapped the reads against the reference genomes of the viruses found to calculate the number of mapped reads, the average read depth, and the percentage of viral genome covered by the reads (Table 2).

**TABLE 1:** Summary of the number of samples collected for melon and weeds in eight different localities surveyed in Spain

Site	Melon	*E. elaterium*	*S. nigrum*	*M. sylvestris*
Site 1 (Cartagena, Murcia)	10	7	7	7
Site 2 (Cartagena, Murcia)	11	7	7	7
Site 3 (Torre-Pacheco, Murcia)	12	7	7	5
Site 4 (Lo Jurado, Murcia)	15	16	0	0
Site 5 (Aguilas, Murcia)	12	0	0	0
Site 6 (Santomera, Murcia)	0	10	10	10
Site 7 (Villamanrique, Castilla-La Mancha)	10	8	7	0
Site 8 (Villaconejos, Madrid)	12	7	0	7
Total	82	62	38	36

**TABLE 2:** List of viruses identified in melon and associated weeds by HTS

Reference ID	Virus	Acronym	Segment	Virus length	MP^*a*^	AD^*b*^	PC^*c*^
Melon							
MN398900	Cucumis melo alphaendornavirus	CmEV		15,084	111,371	868.23	99.95
NC_002034	Cucumber mosaic virus	CMV	RNA1	3,357	591,932	7,573.84	99.70
NC_002035			RNA2	3,050	863,649	7,620.99	99.70
NC_001440			RNA3	2,216	1,888,683	7,581.22	100.00
NC_001504	Melon necrotic spot virus	MNSV		4,266	6,024	187.98	99.08
NC_001556	Tobacco mild green mosaic virus	TMGMV		6,355	128	3.52	66.89
NC_003688	Cucurbit aphid -borne yellows virus	CABYV		5,669	351,189	6,478.45	99.82
NC_004611	Tomato leaf curl New Delhi virus	ToLCNDV	DNA-A	2,739	69,863	2,902.98	100.00
NC_004612			DNA-B	2,696	7,828	450.81	74.03
NC_006262	Watermelon mosaic virus	WMV		10,035	2,728,171	7,428.15	99.30
NC_018173	Cucurbit chlorotic yellows virus	CCYV	RNA1	8,607	1,501	21.15	99.17
NC_018174			RNA2	8,041	3,893	62.21	99.63
*E. elaterium* (yr 2020)							
NC_001556	Tobacco mild green mosaic virus	TMGMV		6,355	722	13.64	76.74
NC_003688	Cucurbit aphid borne yellows virus	CABYV		5,669	631,106	6,570.95	99.29
NC_003743.1	Turnip yellows virus	TuYV		5,641	56,381	2,307.51	30.79
NC_004611	Tomato leaf curl new Delhi virus	ToLCNDV	DNA-A	2,739	131,442	4,174.19	100.00
NC_004612			DNA-B	2,696	31,148	1,733.61	75.37
NC_004809.1	Cucurbit yellow stunting disorder virus	CYSDV	RNA 1	9,123	5,270	76.00	99.25
NC_004810.1			RNA 2	7,976	13,720	210.78	99.68
*E. elaterium* (yr 2021)							
NC_001556	Tobacco mild green mosaic virus	TMGMV		6,355	504	6.66	5.28
NC_003688	Cucurbit aphid borne yellows virus	CABYV		5,669	667,898	14,077.18	98.00
NC_003743.1	Turnip yellows virus	TuYV		5,641	127,479	1,859.56	28.32
NC_004611	Tomato leaf curl new Delhi virus	ToLCNDV	DNA-A	2,739	244,070	10,825.60	100.00
NC_004612			DNA-B	2,696	52,938	1,928.22	75.29
NC_004809.1	Cucurbit yellow stunting disorder virus	CYSDV	RNA 1	9,123	38,161	548.77	98.85
NC_004810.1			RNA 2	7,976	108,553	1,729.99	99.72
*S. nigrum*							
NC_001616	Potato virus Y	PVY		9,704	977,131	6,166.02	98.80
NC_003828.1	Tomato yellow leaf curl Sardinia virus	TYLCSV		2,773	142	5.62	61.37
NC_020073.2	Moroccan pepper virus	MPV		4,772	223	7.24	80.28
NC_034240	Tomato yellow mottle associated virus	ToYMaV		13,389	18,933	665.20	9.45
*M. sylvestris*							
MN116683.1	Malva vein clearing virus	MVCV		9,659	661,235	5,939.87	96.38
NC_001616	Potato virus Y	PVY		9,704	728	10.36	81.20
NC_011058	Chickpea chlorotic dwarf virus	CpCDV		2,584	5,883	324.41	65.78
NC_025389	Eggplant mottled dwarf virus	EMDV		13,100	23,700	220.00	91.71
NC_034240	Tomato yellow mottle-associated virus	ToYMaV		13,389	145	7.78	3.89

a Number of reads mapped (MP) to the virus genome.

b Average depth (AD) of reads mapped to the virus genome.

c Percentage (PC) of virus genome covered by the reads.

### Viruses infecting melon.

We detected eight viruses in melon samples (Table 2). Five of these, cucumber mosaic virus (CMV), melon necrotic spot virus (MNSV), CABYV, WMV, and ToLCNDV, were already reported infecting melon in Spain (13, 28, 29). In addition, we found TMGMV, CCYV, and CmEV for the first time in melon in Spain. CMV sequences were the most abundant in our data set with around 4 M reads, followed by WMV (around 2.7 M reads), then CABYV (0.3 M reads), and CmEV (0.1 M reads) (Table 2). The less abundant sequences in our data set corresponded to CCYV (5,394 reads) and TMGMV (128 reads). Although the number of reads corresponding to TMGMV was low, the percentage of the viral genome covered by reads was high (66.9%) (Table 2). To determine the occurrence of these viruses in individual melon samples, we used quantitative RT-PCR (qRT-PCR) with specific primers and probes (Table S2). The results are summarized in Table 3. CmEV was present in 100.0% of our samples (Table 3). Next, WMV was detected in 87.8% of the samples, closely followed by CABYV, which infected 74.4% of the samples, and ToLCNDV, which was detected in 62.2% of the samples. CMV had a frequency of 26.8%. We found that MNSV, TMGMV, and CCYV infected less than 16.0% of the samples. Our results showed no correlation between the abundance of viral reads in the sequencing data from melon (Table 2) and the proportion of plants infected by a given virus (Table 3), probably reflecting large differences in virus accumulation within host plants.

**TABLE 3:** Summary of specific virus detection in melon and *E. elaterium* samples

	Positive samples (total melon samples per site)									
	Melon								*E. elaterium*	
Site	MNSV	CMV	TMGMV	CCYV	WMV	CmEV	CABYV	ToLCNDV	CABYV	ToLCNDV
Site 1	1 (10)	2 (10)	4 (10)	0 (10)	1 (10)	10 (10)	10 (10)	0 (10)	6 (7)	1 (7)
Site 2	7 (11)	0 (11)	0 (11)	1 (11)	10 (11)	11 (11)	9 (11)	9 (11)	7 (7)	7 (7)
Site 3	0 (12)	0 (12)	0 (12)	0 (12)	12 (12)	12 (12)	12 (12)	12 (12)	6 (7)	7 (7)
Site 4	0 (15)	2 (15)	0 (15)	0 (15)	15 (15)	15 (15)	15 (15)	15 (15)	7 (16)	16 (16)
Site 5	5 (12)	6 (12)	4 (12)	0 (12)	12 (12)	12 (12)	12 (12)	12 (12)	NA	NA
Site 6	NA	NA	NA	NA	NA	NA	NA	NA	2 (10)	8 (10)
Site 7	0 (10)	1 (10)	0 (10)	0 (10)	10 (10)	10 (10)	0 (10)	1 (10)	2 (8)	3 (8)
Site 8	0 (12)	11 (12)	0 (12)	0 (12)	12 (12)	12 (12)	3 (12)	1 (12)	2 (7)	7 (7)
Total	13 (82)	22 (82)	8 (82)	1 (82)	72 (82)	82 (82)	61 (82)	51 (82)	32 (62)	49 (62)
Percentage^*a*^	15.85%	26.80%	9.70%	1.21%	87.80%	100%	74.40%	62.20%	51.61%	79.03%

a Percentage of infected samples (infected samples/total melon samples × 100).

### Viruses infecting weeds.

We then explored the virome of weeds in the margins of melon crops to detect potential sources of infection for melon. In *E. elaterium* we detected five viruses; CABYV was the most abundant, with 0.6 M reads mapping its genome, followed by ToLCNDV, supported by 0.2 M reads for both DNA-A and DNA-B (Table 2). The viruses with a lower number of mapping reads were cucurbit yellow stunting disorder virus (CYSDV) (18,990 reads for both RNA1 and RNA 2) and TMGMV (722 reads) (Table 2). The percentages of genome coverage for these less abundant viruses were, however, very high, 99.3% for CYSDV and 76.9% for TMGMV (Table 2). In addition, we recovered a contig of 2,629 nt that showed 77.8% identity with turnip yellows virus (TuYV) (Table S3); the number of mapping reads to TuYV were 56,381 with a genome coverage of only 30% (Table 2). This sequence seems to be very divergent from TuYV and may constitute a partial sequence of a new virus species.

We detected four viruses in *S. nigrum*: potato virus Y (PVY), tomato yellow leaf curl Sardinia virus (TYLCSV), Moroccan pepper virus (MPV), and a sequence related to tomato yellow mottle-associated virus (ToYMaV) (Table 2). The most abundant in this data set was PVY with 0.9 M reads (Table 2). MPV and TYLCSV were the less abundant with 223 and 142 reads, respectively (Table 2). Even with a small number of reads, the percentages of their genome coverage were high for MPV (80.3%) and TYLCSV (61.4%) (Table 2). The sequence related to ToYMaV was a contig of 3,786 nucleotide (nt), which shared 67.0% nt identity with this virus (Table S3); the reads mapping and the genome coverage to ToYMaV were low (18,933 reads and 9.5%, respectively), suggesting that this sequence could belong to the partial genome of a new cytorhabdovirus.

In the case of *M. sylvestris*, we detected malva vein clearing virus (MVCV), supported by 0.6 M reads, followed by eggplant mottled dwarf virus (EMDV) with 23,700 reads, and chickpea chlorotic dwarf virus (CpCDV) with 5,883 reads (Table 2); to our knowledge this is the first report of CpCDV in Spain. PVY was also detected in malva, although with only 728 reads. The percentage of reads covering these viruses’ genomes was high (greater than 65.8%). Interestingly, we got a partial sequence of 2,082 nt in length (Table S3) showing 65.0% identity with ToYMaV (Table S3). The number of reads mapped to ToYMaV and its coverage (145 reads and 3.9% of coverage, respectively) further suggested the presence of a new putative cytorhabdovirus (Table 2). Pairwise identity between the two partial new cytorhabdovirus sequences from *S. nigrum* and *M. sylvestris* yielded a value of 95.2%. In addition, we detected the near complete genomes of three new plant viruses in the weeds data set: *Soymovirus masolus* in *M. sylvestris* (30) and two additional viruses which will be reported elsewhere.

### *E. elaterium* as a virus reservoir for viruses infecting melon.

Among the weeds analyzed, *E. elaterium* was the only species that shared viruses (CABYV, ToLCNDV and TMGMV) with melon, suggesting its possible role as a virus reservoir for the crop species. Given the potential importance of *E. elaterium* in the epidemiology of melon viruses, we first resampled *E. elaterium* during the 2021 season in three sites (Table S4) and sequenced the pooled samples to confirm its virome (Table S1). We detected the same viruses as in 2020 (Table 2). Briefly, CABYV was the most abundant in our new data set (0.6 M reads belonged to its genome), followed by ToLCNDV with 0.4 M reads mapping to both DNA-A and DNA-B covering more than 75.0% of its genome. CYSDV and TMGMV were less abundant in the new data set (less than 56,000 reads). We detected again the putative new virus related to TuYV (see above).

The relevance of CABYV and ToLCNDV as melon pathogens is well known, but this is not the case for TMGMV, the third virus shared between *E. elaterium* and melon. Therefore, we next studied the TMGMV potential to infect and cause disease in melon. Mechanical inoculations of melon plants suggested that TMGMV can indeed infect melon (Table S5). No disease symptoms were recorded in infected plants during an observation period of 20 days’ postinoculation. Therefore, the detection of TMGMV in the HTS data set may reflect true infections, although the relevance of this virus in disease induction in melon is likely negligible.

Lastly, we studied the incidence of CABYV and ToLCNDV in *E. elaterium* during the 2020 season. We extracted total RNA from individual samples and screened them for the presence of the two viruses using RT-PCR. We detected CABYV in 32 out of 62 samples (51.6%) from the 7 sites analyzed (Table 3). The percentage of infected samples varied among the different sites and ranged from 20.0% to 28.6% for sites 6, 7, and 8, and from 43.7% to 100.0% for sites 1 to 4 (Table 3). In the case of ToLCNDV, 49 out of 62 samples (79.0%) were positive (Table 3). We found ToLCNDV-infected samples in the seven sites surveyed, and the percentage of infection by site ranged from 14.3% in site 1, to 100.0% in sites 2, 3, 4 and 8. Therefore, like for melon, the incidence of CABYV and ToLCNDV in *E. elaterium* was high. All in all, the above data reinforce the idea of *E. elaterium* as a potential alternative host and reservoir of CABYV and ToLCNDV for melon crops. The testing of this hypothesis is described in the following sections.

### Phylogenetic relationships between CABYV isolates.

**FIG 2:**
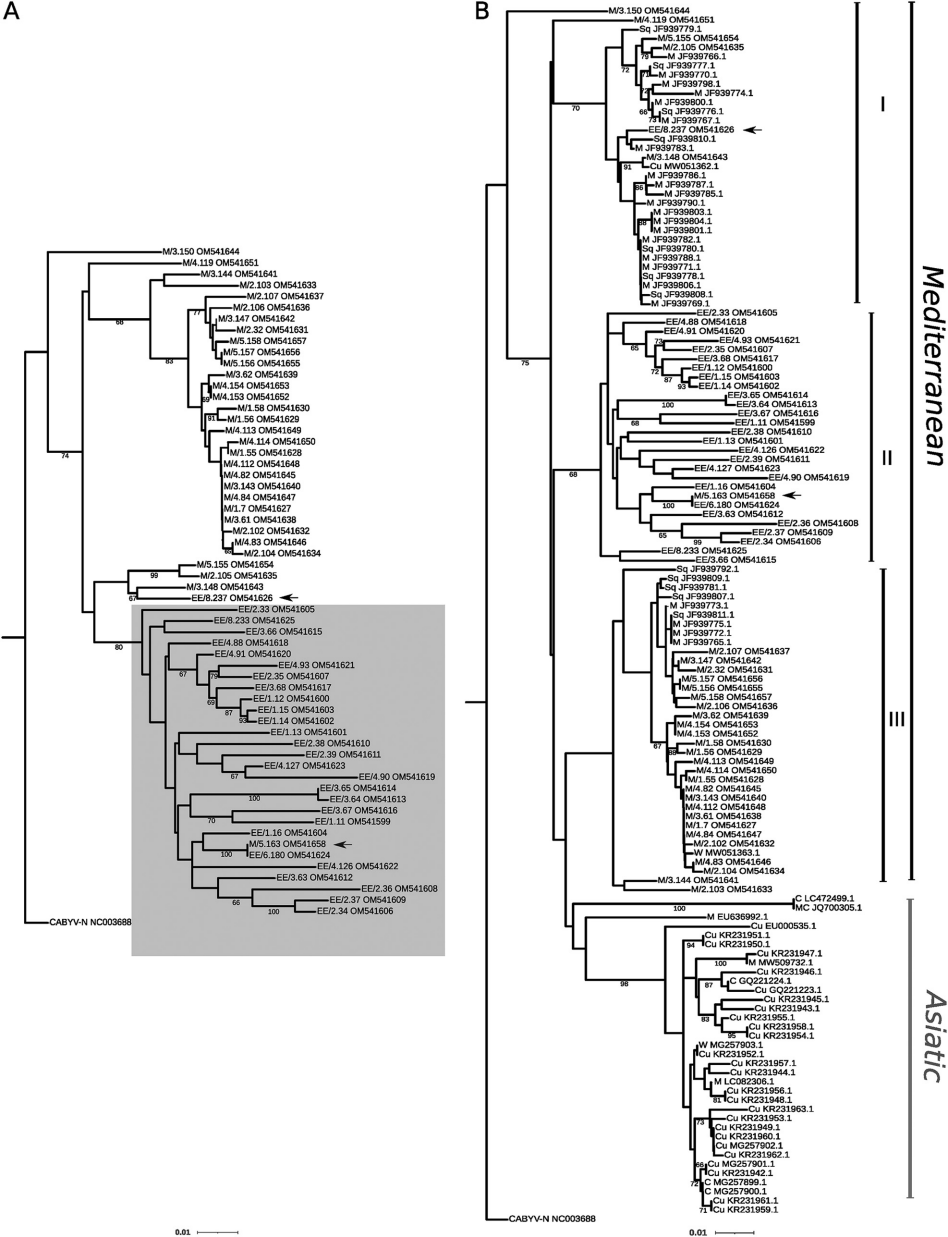
Phylogenetic relationships between different isolates of cucurbit aphid-borne yellows virus (CABYV) inferred from partial sequences of open reading frame (ORF) 2. Minimum evolution trees were inferred using the MEGA X program using the K2P substitution model following a Gamma distribution. A first tree was inferred using 60 Spanish isolates characterized in this study (A); the cluster formed by CABYV isolates from *E. elaterium* is highlighted in gray. The sequence of a French isolate, CABYV-N (NC003688), was used as an outgroup. A second tree was inferred by incorporating a total of 86 CABYV isolates from Asia and Spain downloaded from the NCBI database (B). The arrows show host-specific intercrossing. First letters denote the host species: M, melon; EE, *E. elaterium*; W, watermelon; C, *Cucumis sativus*; Cu, *Cucurbitaceae*; MC, *Momordica charantia*; Sq, squash. The number in the format X.Y indicates the site of collection and sample number (e.g., M/3.150 is sample 150 collected from site 3). The scale bar corresponds to 0.01 nucleotide substitutions per site. Bootstrap values (1,000 pseudoreplicates) > 65% are shown.

We cloned and Sanger-sequenced a genomic fragment of the CABYV ORF 2 comprising 11.6% of the CABYV genome. This genomic region holds the largest sequence variability for CABYV (18). We sequenced 60 clones, with 1 cDNA clone per infected sample. After aligning the sequences, a recombination analysis was carried out; no likely recombination breakpoints were identified (data not shown). Next, we examined the phylogenetic relationships between CABYV isolates (Fig. 2A). A very robust major clade emerged comprising only *E. elaterium* isolates except for one from melon (M/5.163) (Fig. 2A). The phylogeny did not reveal any obvious association with the site of collection.

To examine if the *E. elaterium* clade shares ancestors with older CABYV isolates in the area, or with isolates from geographically distant areas, we included in our analysis CABYV sequences of isolates collected from cucurbit crops in the same geographical area during 2003–2005 (18) and sequences of Asian isolates available in NCBI. The CABYV sequences clearly clustered into two genetic groups, one group including the Asian isolates, and the other clustering the Mediterranean isolates (Fig. 2B). The Mediterranean group was divided into three clades. Clade I grouped mainly cucurbit isolates from the database, whereas clade III grouped melon isolates from this study, which seemed to share ancestors with older melon isolates from the same area (Fig. 2B). Interestingly, clade II grouped *E. elaterium* isolates from this study (Fig. 2B); therefore, as before, this analysis suggests the existence of an *E. elaterium-*specific lineage.

### CABYV population structure and migration fluxes.

We then sought to test any spatial structuring among CABYV populations, thus considering a population as the isolates from both hosts belonging to the same site. According to our analysis of molecular variance (AMOVA), the entire CABYV population was not structured by site, only 2.8% of the variation was associated to the site. In contrast, 97.2% of the variation was found within populations, suggesting that CABYV populations are possibly structured by the host (Fixation index [Fst] = 0.14, *P *<* *0.001). To further confirm these results, we looked for potential correlations between geographic and genetic distances among pairs of isolates, but no significant correlation was detected (Mantel test, *P* > 0.1).

**FIG 3:**
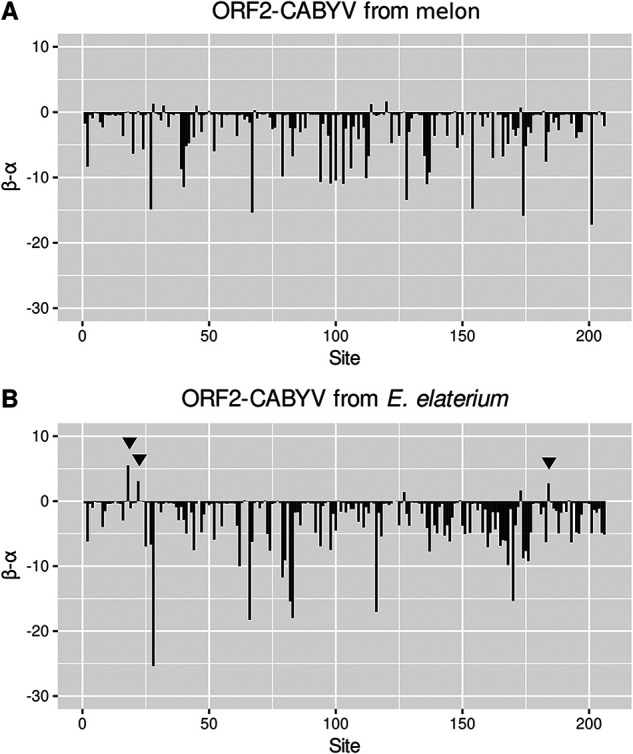
Genetic diversity of cucurbit aphid-borne yellows virus (CABYV) isolates in mean posterior nonsynonymous (β) and synonymous (α) positions at each codon of the open reading frame (ORF) 2 for isolates from melon (A) and *E. elaterium* (B). Differences between the estimated diversity values (β-α) are represented in the horizontal axis. Lines marked with arrows represent sites under positive selection detected by the FUBAR method implemented in the Datamonkey web server with a posterior probability value of 0.9.

We next estimated the genetic diversity (π) for the CABYV population. The total nucleotide diversity of CABYV was 0.0649 ± 0.0067. The nucleotide diversity of the CABYV isolates from *E. elaterium* was very similar to that found for the entire population and higher than that of the melon isolates (0.0621 ± 0.0063 and 0.0337 ± 0.0042, respectively). We next examined if this difference was maintained across the different sites. To do so, we only considered sites 1, 2, 3, and 4, where CABYV was present in both melon and *E. elaterium*. For this analysis, we considered a CABYV subpopulation as the isolates belonging to the same host in each of the four sites. Overall, the nucleotide diversity of the CABYV subpopulations from *E. elaterium* was higher than in melon across the different sites (Table 4). In site 1, the nucleotide diversity was 0.0592 for CABYV from *E. elaterium* and 0.0085 for CABYV from melon; this difference was similar in site 4 (0.0551 and 0.0143, respectively), but lower differences were observed for sites 2 and 3 (0.0608 and 0.0381, and 0.0615 and 0.0428, respectively) (Table 4). We next estimated the diversity in synonymous (dS) and nonsynonymous (dN) positions of the coding sequences to infer the direction and strength of the selection pressure. For all subpopulations, the dN-dS difference was below 0 (Table 4), suggesting that ORF 2 was under purifying selection in both hosts. To analyze this aspect in more detail, we evaluated the codons under selection, finding no significant evidence of positive selection in ORF 2 codons for CABYV from melon (Fig. 3A). In contrast, three codons (positions 18, 22, and 184) were found to be significantly under positive selection in ORF 2 for CABYV from *E. elaterium* (Fig. 3B). Moreover, we found more codons under negative selection in *E. elaterium* compared to melon (58 sites versus 36 sites) (Tables S6 and S7), and a different distribution pattern of codons under selection (Fig. 3). An alignment of the CABYV amino acid sequences around the three codons subjected to positive selection in *E. elaterium* showed that glutamic acid coexisted with lysine in position 18, valine coexisted with glutamic acid, isoleucine, and aspartic acid at position 22, and aspartic acid coexisted with asparagine at position 184 (Fig. S2). The above results suggest that host adaptation could be contributing to genetic differentiation of the CABYV population.

**TABLE 4:** Genetic diversity parameters estimated for between and within CABYV and ToLCNDV subpopulations

		CABYV				ToLCNDV			
Site	Host	π^*a*^ ± SE	dS^*b*^ ± SE	dN^*c*^ ± SE	dN-dS^*d*^	π^*a*^ ± SE	dS^*b*^ ± SE	dN^*c*^ ± SE	dN-dS^*d*^
Site 1	*E. elaterium*	0.0592 ± 0.0077	0.1797 ± 0.0250	0.0120 ± 0.0038	**−**0.1677				
	Melon	0.0085 ± 0.0029	0.0104 ± 0.0069	0.079 ± 0.0033	**−**0.0686				
Site 2	*E. elaterium*	0.0608 ± 0.0074	0.1786 ± 0.0234	0.0127 ± 0.0036	**−**0.1659	0.0036 ± 0.0016	0.0065 ± 0.0051	0.0025 ± 0.0020	**−**0.004
	Melon	0.0381 ± 0.0059	0.1013 ± 0.0183	0.0112 ± 0.0033	**−**0.0901	0.0034 ± 0.0020	0.0000 ± 0.0000	0.0047 ± 0.0029	0.0047
Site 3	*E. elaterium*	0.0615 ± 0.0077	0.1701 ± 0.0251	0.0179 ± 0.0046	**−**0.1522	0.0038 ± 0.0018	0.0056 ± 0.0033	0.0031 ± 0.0024	**−**0.0025
	Melon	0.0428 ± 0.0058	0.01181 ± 0.0196	0.0096 ± 0.0032	**−**0.0022	0.0040 ± 0.0020	0.0021 ± 0.0022	0.0048 ± 0.0028	0.0027
Site 4	*E. elaterium*	0.0551 ± 0.0070	0.1473 ± 0.023	0.0135 ± 0.0041	**−**0.1338	0.0036 ± 0.0018	0.0018 ± 0.0018	0.0049 ± 0.0028	0.0031
	Melon	0.0143 ± 0.0028	0.0370 ± 0.0104	0.0049 ± 0.0018	**−**0.0321	0.0038 ± 0.0018	0.0066 ± 0.0051	0.0036 ± 0.0023	**−**0.0030

a Nucleotide diversity (mean nucleotide differences per site between sequence pairs).

b Frequency of synonymous substitution per site.

c Frequency of nonsynonymous substitution per site.

d Difference between dN and dS.

**FIG 4:**
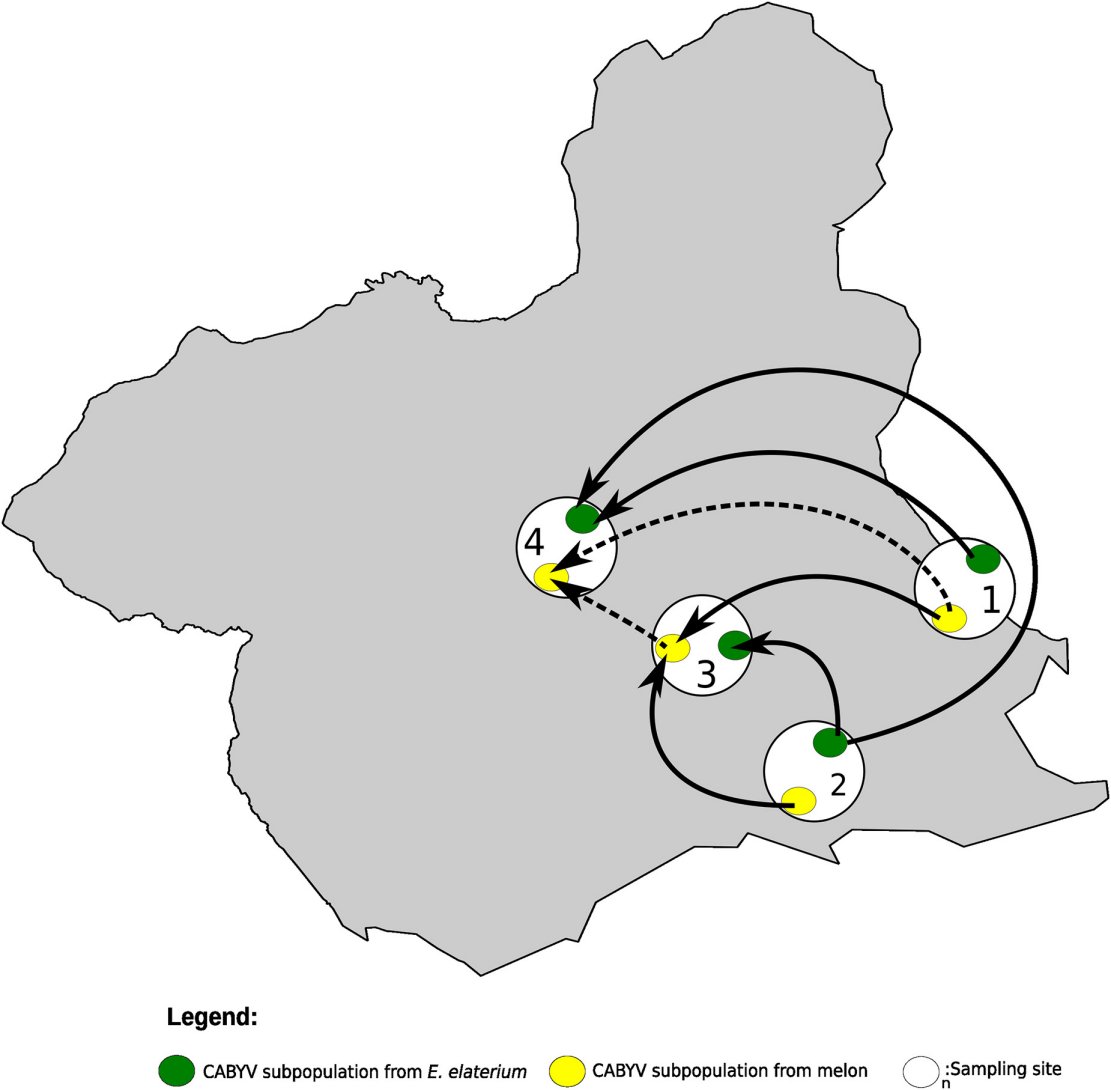
Diffusion pathway of Cucurbit aphid-borne yellows virus (CABYV) among melon crops and crop edge weeds in sites 1, 2, 3, and 4. Thickness of lines represents supported migration rates with a mean indicator of >0.5: solid black arrows, very strong support with BF >15; dashed black arrows, strong support 3 < BF < 15. The Murcia region (Spain) is shown in gray. White circles show the sites from where CABYV was isolated from both hosts, melon and *E. elaterium*. Yellow and green circles represent CABYV populations from melon and *E. elaterium*, respectively, in each of the sites.

We then investigated genetic differentiation using the Fst parameter. Fst values among host subpopulations within sites ranged from 0.35 to 0.61 (Table 5), suggesting differentiation between the *E. elaterium* and melon sequences within each site. We then examined the CABYV differentiation among geographical subpopulations of the same host, either melon or *E. elaterium*. In melon, the Fst values ranged from 0 to 0.18 (Table 5), suggesting nondifferentiated subpopulations. In *E. elaterium*, Fst values were lower than 0.07 (Table 5), suggesting undifferentiated subpopulations as well. To study potential diffusion of CABYV across the four sites, we reconstructed the CABYV possible migration pathways using Bayesian methods. Results showed seven likely migration events with mean migration rates ranging from 0.99 to 1.83 (Table S8); migrations with the higher scores where from CABYV subpopulations in *E. elaterium* from site 1 and 2 to *E. elaterium* in site 4 (Fig. 4) and from melon in site 1 to melon in sites 3 and 4 (Fig. 4). Importantly, the migration patterns in these four sites were between *E. elaterium* or melon subpopulations, but not for subpopulations across the two different hosts (Table S8; Fig. 4).

**TABLE 5:** Degree of differentiation (Fst) between and within CABYV subpopulations

CABYV									
Site 1		Site 2		Site 3		Site 4			
*E. elaterium*	Melon	*E. elaterium*	Melon	*E. elaterium*	Melon	*E. elaterium*	Melon	Host	Site
	0.61	0.07	0.43	0.06	0.39	0.04	0.56	*E. elaterium*	Site 1
		0.60	0.18	0.59	0.18	0.6	0.05	Melon	
			0.42	0.03	0.40	0.05	0.56	*E. elaterium*	Site 2
				0.40	0.00	0.43	0.15	Melon	
					0.35	0.06	0.54	*E. elaterium*	Site 3
						0.38	0.13	Melon	
							0.57	*E. elaterium*	Site 4
								Melon	

Ultimately, our genetic population and migration analyses agreed with our phylogenetic observations, strongly suggesting that there are at least two genetic groups of CABYV in the Murcia region, one infecting melon, and the other infecting *E. elaterium*, with unlikely migration events between hosts but frequent exchange within hosts.

### Phylogenetic relationships between ToLCNDV isolates.

**FIG 5:**
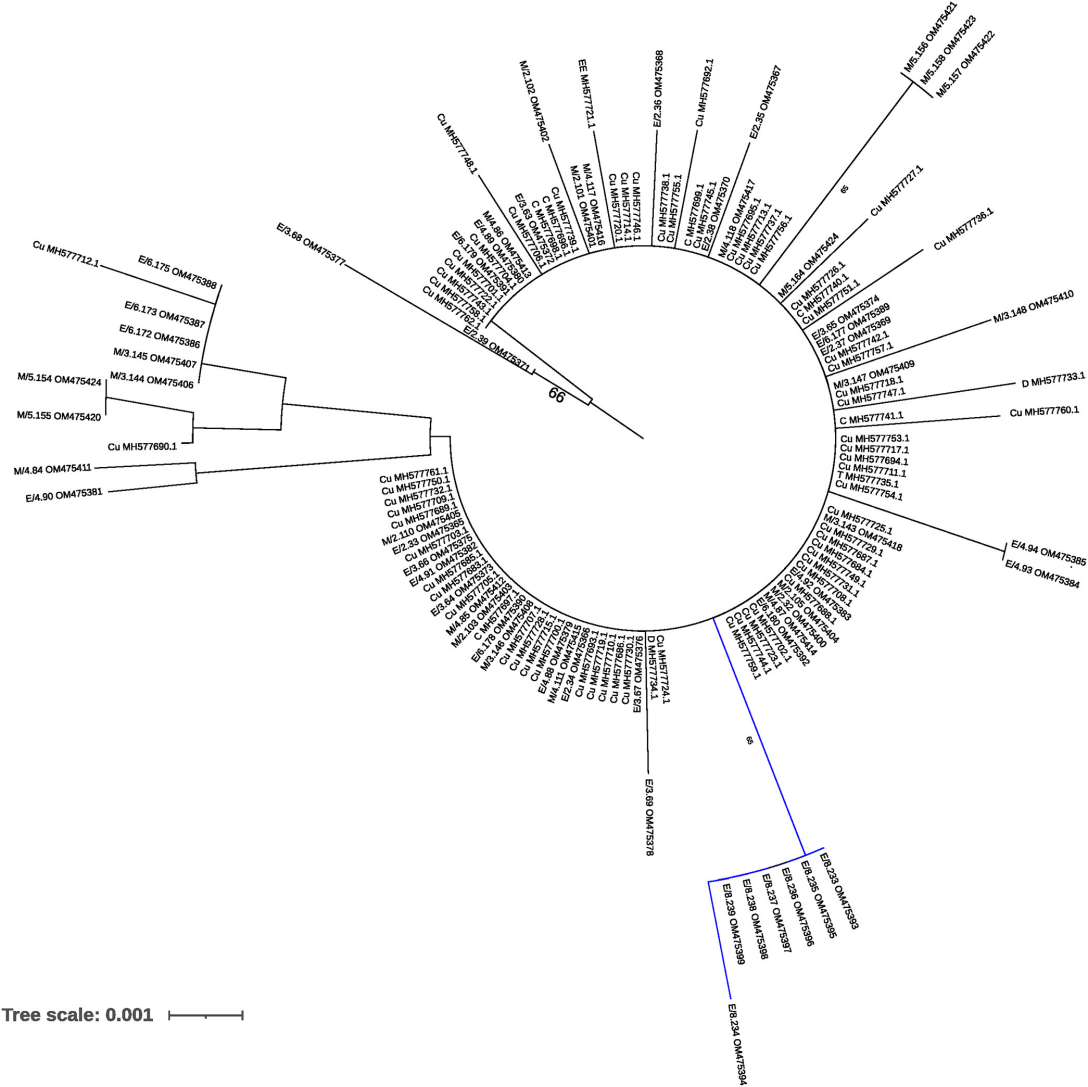
Phylogenetic tree of Spanish tomato leaf curl New Delhi virus (ToLCNDV) isolates inferred from the full sequence of the AV2 gene. Minimum evolution trees were inferred from an alignment incorporating the 60 isolates from this study and another 77 Spanish isolates from the NCBI database with the MEGA X program using the K2P substitution model. First letters denote the host species: M, melon; EE, *E. elaterium*, C: *Cucumis sativus*; Cu, Cucurbitaceae; D, Datura stramonium and T, Tomato. The number in the format X.Y indicates the collection site and the sample number (e.g., M/3.150 is sample 150 collected from site 3). The scale bar corresponds to 0.001 nucleotide substitutions per site. Bootstrap values (1,000 pseudoreplicates) > 65% are shown.

We cloned the complete AV2 gene from 60 ToLCNDV isolates, with 1 clone sequenced per infected sample. This covered 17.3% of the ToLCNDV DNA-A genome. Our preliminary analysis suggested very little diversity (see below), so the branching of the phylogenetic tree that we built for the isolates in this work had a weak significance (data not shown). Adding isolates from databases strengthened the tree’s significance, and, thus, the analysis illustrated in Fig. 5 includes the 60 isolates from this study and another 77 Spanish isolates downloaded from NCBI. Our phylogenetic analysis showed a homogeneous Spanish ToLCNDV population and excluded any obvious association to the host (Fig. 5); however, we spotted one group that clustered apart from the rest of the isolates with a high bootstrap value that gathered the isolates from site 8, suggesting that the collection site may play a role in the genetic differentiation of the ToLCNDV populations.

### ToLCNDV population structure and migration fluxes.

**FIG 6:**
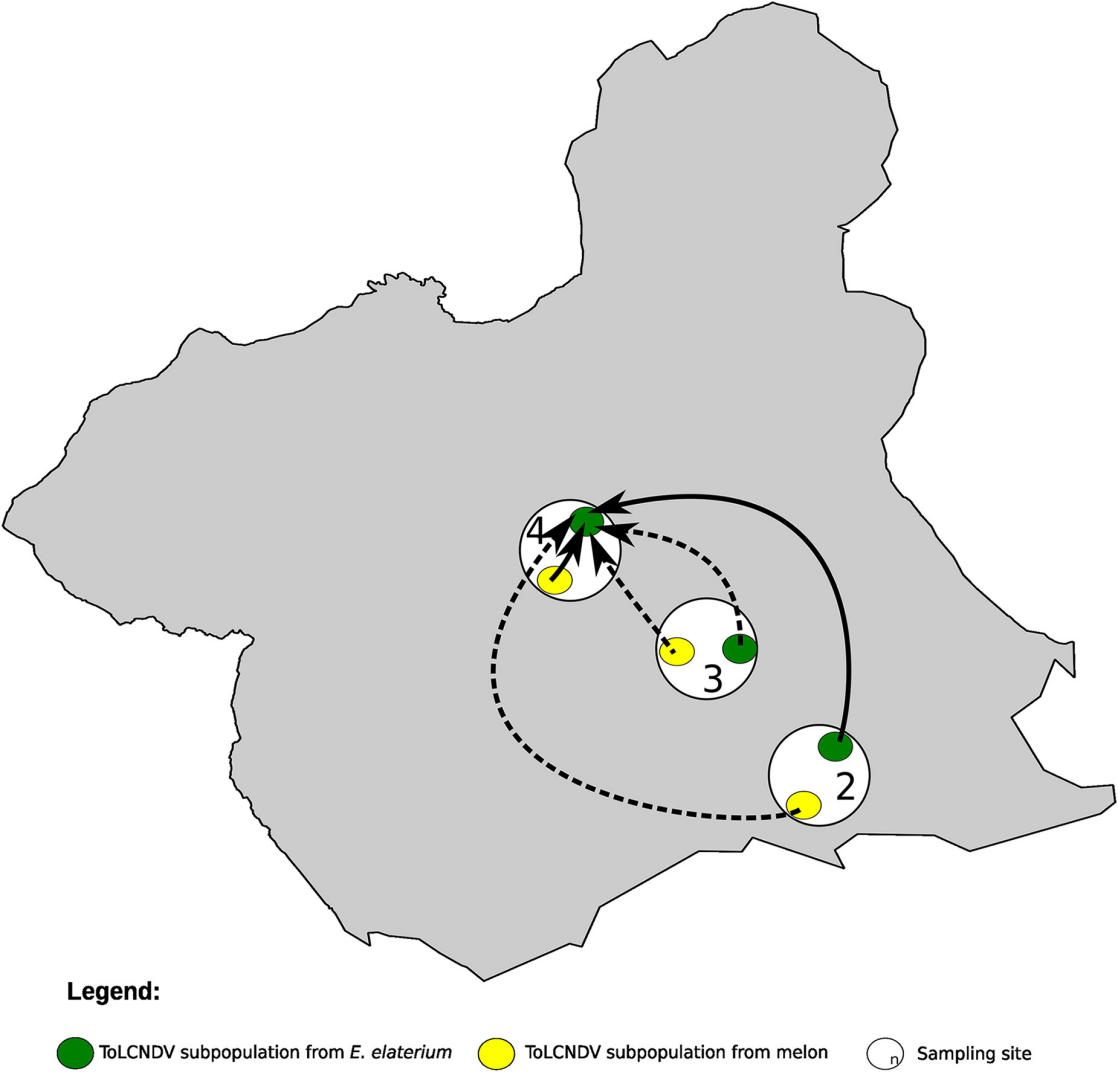
Diffusion pathway of Tomato yellow leaf curl New Delhi virus (ToLCNDV) among melon crops and crop edge weeds in sites 2, 3, and 4. Thickness of lines represents supported migration rates with a mean indicator of >0.5: solid black arrows, very strong support with BF >15; dashed black arrows, strong support 3 < BF < 15. The Murcia region (Spain) is shown in gray. White circles show the sites from where ToLCNDV was isolated. Yellow and green circles represent ToLCNDV populations from melon and *E. elaterium*, respectively, in each of the sites.

Considering a population as the isolates from any host belonging to the same site, we performed an AMOVA test and found that 26.3% of the variation was associated to the site (Fst = 0.26 and *P *<* *0.001). To further test this hypothesis, we sought to identify potential correlations between geographic and genetic distances. We found a correlation of 0.8 between the genetic and geographic matrices (Mantel test, *P* < 0.05), suggesting a potential subgrouping according to geographical sites; nevertheless, we found that the nucleotide diversity for the entire population, and the nucleotide diversity within sites, were very low (Table 4). In fact, the nucleotide diversity for the entire ToLCNDV population was 0.0045 ± 0.0013, more than 1 order of magnitude below that found for the CABYV population (see above). We next analyzed ToLCNDV genetic differentiation between and within sites 2, 3, and 4, where ToLCNDV infected both melon and *E. elaterium*. Within subpopulations, the Fst values were lower than 0.09 (Table 6); between subpopulations, the Fst values ranged from –0.02 to 0.09 (Table 6), showing no differentiation. Since our phylogeny suggested a subgrouping of ToLCNDV isolates from site 8 compared to the rest (Fig. 5), we investigated if ToLCNDV is differentiated in site 8 in comparison to ToLCNDV from sites 2, 3, and 4. Since site 8 had ToLCNDV only in *E. elaterium*, we considered just this host species for the four sites. The Fst values ranged from 0.58 to 0.72, suggesting differentiation of this subpopulation compared to the other sites (Table 6). As for CABYV, we used Bayesian methods to infer migration among ToLCNDV subpopulations. We identified six likely migration events with the mean migration rates ranging from 0.96 to 1.34 (Table S9); migrations were from melon to *E. elaterium* within sites (site 4; Table S9 and Fig. 6) and between different sites (from melon in sites 2 and 3 to *E. elaterium* in site 4; Table S9 and Fig. 6). Interestingly, migrations with high scores were all from melon to *E. elaterium*. Altogether, our results showed that melon is a likely source of infection of ToLCNDV for *E. elaterium* and also suggested a possible role of geographic distribution in ToLCNDV diversification.

**TABLE 6:** Degree of population differentiation (Fst) between and within ToLCNDV subpopulations

ToLCNDV								
Site 2		Site 3		Site 4		Site 8		
*E. elaterium*	Melon	*E. elaterium*	Melon	*E. elaterium*	Melon	*E. elaterium*	Host	Site
	0.09	**−**0.02	0.00	0.03	**−**0.01	0.63	*E. elaterium*	Site 2
		0.06	0.09	0.09	0.00	0.49	Melon	
			0.00	−0.02	−0.05	0.58	*E. elaterium*	Site 3
					−0.01	0.45	Melon	
					0	0.61	*E. elaterium*	Site 4
						0.46	Melon	
							*E. elaterium*	Site 8

## DISCUSSION

We have used HTS to characterize the viromes of melon and three associated weed species sampled within two geographical areas in 2020 in Spain. *E. elaterium*, a cucurbit weed, emerged as a potential alternative host for melon viruses. Given its permanence throughout cropping seasons, we decided to study its potential role as virus reservoir for melon crops. Cloning and Sanger sequencing of CABYV and ToLCNDV isolates were used in phylogenetic and population genetics analyses, identifying a CABYV lineage specific for *E. elaterium*. Further analyses were conducted to examine possible migrations of CABYV and ToLCNDV between *E*. *elaterium* and melon within and between the different sampling sites.

Melon is one of the main horticultural crops in Spain, and viral diseases can threaten its production. Among the different viruses reported to infect melon (28, 29, 31, 32), CABYV seems to be the most prevalent virus followed by WMV (7, 15, 18). In our case, we found that WMV was the prevalent virus in melon crops followed by CABYV; this discrepancy may be due to an actual change in the relative prevalence of the viruses in the area, but also to the sample size or even to the different methods used for detection, perhaps with different sensitivities for the two viruses. In any case, the high occurrence of both viruses in the area is likely related to epidemiological factors including transmission or the existence of virus reservoirs associated to alternative hosts. Our weed virome analysis was useful in this respect, identifying *E. elaterium* as a host for CABYV and ToLCNDV, but not for WMV. Given the broad WMV host range, other weeds not covered in this study might host this virus; alternatively, a deeper sampling effort in *E. elaterium* may identify this species as a natural host for WMV although at low prevalence. ToLCNDV was the third most frequent virus in our data set; since its introduction around 2012, its incidence has become very important in cucurbit crops (8). In addition, we reported the presence of CmEV, TMGMV, and CCYV for the first time in melon crops in Spain. CmEV was described for the first time in melon in 2016 (33) and later reported in China (34). This virus is transmitted vertically, and its effects and potential symptomatology in the infected plants remain unexplored. Surprisingly, its incidence was 100% in our melon samples. This may be explained by a high incidence in the melon germplasm used in Spain, as it appears to be the case in the United States (33). TMGMV has been reported in different crops and countries (35–39), but never in melon; our data suggest that indeed it was infecting the melon samples, although its epidemiological relevance is possibly negligible. We also reported CCYV for the first time in melon in Spain. CCYV was first reported in Japan (40) and then in Greece in 2011 (41) and in Egypt (42) and Saudi Arabia (43) in 2014. Recently, it was detected in the South of Spain in cucumber (29); its recent introduction could explain its low incidence in our samples. Broader surveys should be conducted to assess its prevalence and spread among the different cucurbit production areas.

The role of weeds in the occurrence and spread of plant viruses is an integral part of disease epidemiology. Numerous reports (44–47) have assessed the role of weeds as host and reservoirs for viruses that infect crops. In our case, we found an overlap between the *S. nigrum* and *M. sylvestris* viromes, and the viruses identified in these hosts may have significant agronomic importance for certain crops, although not for cucurbits. *E. elaterium* shared the most viruses with melon likely due to its phylogenetic proximity to melon. Both CABYV and ToLCNDV were already reported to infect these two plant species (8, 18).

Since *E. elaterium* was the only weed sharing its virome with melon, one hypothesis we sought to test was its potential to act as a source of inoculum for melon crops, which would imply the sharing of virus genotypes between the two host species. In contrast, we found evidence of genetic differentiation of CABYV subpopulations infecting melon and *E. elaterium* and little differentiation of geographically distant CABYV subpopulations within each host. These conclusions were further strengthened by our migration analysis, which suggested frequent virus migration among sites within each host, but unlikely migration events between hosts, even if plants of the two species were in the same site. One explanation for the strong influence of the host on the structure of CABYV populations could be the existence of ecological barriers preventing transmission of the virus between plants of the different host species (48, 49). Such barriers could lead to population differentiation due either to genetic drift or selection. The recovery of viral isolates belonging to heterologous “host-specific” phylogenetic clusters (EE/8.237 in melon and M/5.163 in *E. elaterium*) might provide an indication that barriers to transmission are not absolute and that mechanisms exist for low frequency interspecific transmission (50). CABYV is naturally transmitted by aphids, with Aphis gossypii and Myzus persicae transmitting it in a circulative nonpropagative manner (17, 51). However, CABYV transmission efficiency from/to different hosts has been scarcely studied. Kassem et al. (18) performed CABYV transmission experiments under controlled conditions from *E. elaterium* to melon, showing that, at least under the experimental conditions used, CABYV transmission from the weed to the melon plants could take place (18). Therefore, if barriers exist, they may have a different nature, including that different aphid populations or even different species may thrive in the different hosts in nature. For instance, Rebenstorf et al. (50) reported molecular phylogenetic reconstructions revealing that the diversity of cherry leaf roll virus (CLRV) populations is determined by the host plant from which the viral samples were originally obtained, hypothesizing that this pattern may reflect the exclusive mode of transmission in natural populations by pollen and by seeds. These modes of transmission are likely to impose barriers to host switch, leading to a rapid biological and genetic separation of CLRV variants coevolving with different plant host species (50). We are not aware of modes of CABYV transmission in *E. elaterium* different than aphid vectoring, but this is a hypothesis probably worth to be tested. Moreover, host-specific selection pressures could also lead to the host structuring of CABYV. We evaluated selective constraints in the CABYV sequences by comparing the rates of synonymous and nonsynonymous substitutions across codon sites. Overall, we showed that purifying selection was a major evolutionary force shaping nucleotide diversity of the CABYV coding sequence. Specific amino acid sites were identified as targets of negative selection, with different distribution patterns for both hosts. Also, three cases of positive selection were detected after comparing CABYV sequences from *E. elaterium*; the targeted amino acids may have been selected because of enhanced specificity or enhanced strength of interactions with some host-specific factor(s) (52). In this regard, experiments testing the *in planta* fitness of CABYV isolates in both hosts would be enlightening. Last but not least, another factor that may contribute to the isolation of the CABYV population in *E. elaterium* could be the exclusion of melon isolates by resident *E. elaterium-*adapted isolates. Still, we cannot rule out that all the processes mentioned above may apply simultaneously to contribute to the observed differentiation.

For ToLCNDV, we have described a scenario that contrasts with that for CABYV. Juárez et al. (8) reported that ToLCNDV is widely distributed in cucurbit crops in Spain and that the weeds *E. elaterium*, *D. stramonium*, and *Sonchus oleraceus* can host ToLCNDV; these authors also reported the presence of a monophyletic ToLCNDV population composed of isolates of the ES genotype, with no clustering pattern across hosts, location, or year, although they showed that *D. stramonium* exhibited larger levels of within-population genetic diversity. In agreement with the results mentioned above, the ToLCNDV isolates reported here showed very low genetic diversity without a specific association to the host. Since ToLCNDV has been recently introduced in Spain (28), large variability was not expected. Whiteflies (Bemisia tabaci) transmit the virus in a circulative persistent manner (20). This mode of transmission, together with the high migration potential of the vector within and between different melon growing fields, could contribute to constant virus fluxes that prevent population differentiation. However, and in contrast to what Juárez et al. (8) reported, our results suggest an influence of geography and landscape on potential patterns of ToLCNDV genetic differentiation when enlarging the sampling scale. In site 8 (Villaconejos, Madrid), around 400 Km from the Murcia region, and where melon plants were not infected with ToLCNDV, genetic differentiation was observed when compared to ToLCNDV populations from Cartagena (Murcia). It is likely that the isolation of this population contributed to ToLCNDV differentiation. Moreover, the migration patterns that we estimated for this virus across species were mainly from melon to *E. elaterium*, suggesting that the weed might be a dead-end host for ToLCNDV.

Although further experimental work is needed to validate the hypotheses posed above, the results reported here illustrate very well on the impact that plants in the edges of the fields can have as drivers of the viruses’ diversification and evolution.

## MATERIALS AND METHODS

### Sample collection.

A survey was conducted in open field melon (*Cucumis melo* L.) crops in Spain in 2020. We visited eight sites in total (Fig. 1), six in Murcia, one in Madrid, and one in the Castilla-La Mancha regions, collecting a total of 218 leaf samples from melon and three weeds (Table S4) that are frequently found in borders and edges of melon crops: *E. elaterium*, *S. nigrum*, and *M. sylvestris*. The melon varieties surveyed were “Charentais,” “Piel de Sapo,” and “Yellow melon.” From each site and whenever possible, we randomly collected 10 to 12 melon samples and 7 to 10 from each weed, except for site 4 where we increased the number of plants since the field was large (Table 1). The melon samples collected exhibited virus-like symptoms (yellowing, leaf curling, and mosaics), and weed samples were mostly asymptomatic (Fig. S1 and Table S4). A second-year survey was carried out in the Murcia region. We visited three sites: 3, 4, and 6; leaves from 7 to 16 *E*. *elaterium* plants found in the crop edges were collected. One hundred mg of leaf tissue from each sample was placed in 1.5-mL tubes, frozen and stored. For HTS (see below), 200 mg of leaf material from each sample was kept in 2-mL tubes and used to build the species-site pools; in a first stage, the pools were prepared for each species and site by mixing these 200 mg (Fig. S3). In both cases, tubes were then frozen in liquid N_2_ and stored at −80°C until total RNA extraction.

### Nucleic acid extraction, pooling, and sequencing.

Leaf tissue from each of the species-site pools prepared as described above was ground with a mortar and pestle in the presence of liquid N_2_ until obtaining a fine powder was obtained. Total RNA was extracted using TRI Reagent (Sigma-Aldrich, St. Louis, MO, USA) following the manufacturer’s protocol. Pool preparations were then treated with RNase-free DNase I (New England Biolabs, Ipswich, MA, USA), following the manufacturer’s protocol to remove traces of DNA. The pool preparations were then cleaned up by phenol-chloroform extraction (53), and the RNA integrity was confirmed using gel electrophoresis. The quantity of total RNA was assessed using a Nanodrop One Microvolume UV-Vis Spectrophotometer (Thermo Fisher Scientific, Waltham, MA, USA). Species pools were then prepared by pooling the first pools of the same plant species from different sites to obtain a single tube per species (Fig. S3), by adding the same amount of RNA from each pool. We obtained a final amount of 6 μg of total RNA in a final volume of 60 μL in sterile MilliQ water per sample; samples were sent to Macrogen (Seoul, Korea) for library preparation and sequencing. Four cDNA libraries (one library per species) were synthesized using a TrueSeq Stranded Total RNA sample preparation kit (Illumina, San Diego, CA, USA) with ribosomal depletion using a Ribo-Zero plant kit (Illumina). Sequencing was performed with the Illumina NovaSeq 6000 platform to obtain 150-bp paired end reads. Total RNA from individual melon and *E. elaterium* samples was extracted using the NucleoSpin RNA Plant and Fungi (MACHEREY-NAGEL, Duren, Germany) following the manufacturer’s protocol and used for detection and cloning.

### Bioinformatics analysis.

Raw reads were analyzed using the custom bioinformatics pipeline reported previously (27) with some modifications. The quality of paired-end raw reads was analyzed using the FastQC program. Adapters and low-quality reads (Phred <30) were trimmed from each data set using Trimmomatic v0.39. Paired-end reads were then repaired using BBMap before filtering the host reads (in case of melon) using Bowtie2. Unmapped reads were subjected to de *de novo* assembly using MEGAHIT v1.2.9. Contigs were then aligned against the RefSeq virus database (last updated: Feb 2021) using tblastx. Viral hits were filtered using the following criteria: contig length 0.3 to 15 kb, e-value lower than 10^−4^, and length of alignment between the query and the hit >100 amino acids. The alignment between the query and the hit was further checked manually using Geneious Prime v2020.0.3. RefSeq for each virus identified were used as reference to remap the filtered reads using BWA with the mem algorithm. From these alignments, virus genome coverage and average depth were calculated using SAMtools and R basic functions.

### Virus detection via qRT-PCR.

Viruses were detected in individual samples by qRT-PCR using primers and TaqMan probes designed in this study or reported in previous studies (Table S2). The specificity of the primers and probes was confirmed *in silico* by a blastn search against the NCBI database. The KAPA PROBE FAST One-Step qRT-PCR kit (Roche, Basel, Switzerland) was used. Each 10-μL reaction consisted of 5 μL of 2X Master Mix, 0.2 μL of forward/reverse primers (10 μM) and probe (10 μM), 0.2 μL of 50X RT-Mix, 0.2 μL of 50× ROX high, 2 μL of DEPC-treated water, and 2 μL of RNA (15 ng/μL). The performance of the primers/probe pairs was determined by calculating the PCR amplification efficiency of the reaction from a standard curve of five 1:10 serial dilutions of the pooled total RNA sample (200 ng/μL).

### TMGMV mechanical inoculation.

Melon (cv. Tendral verde tardío) seeds (Fitó seeds, Barcelona, Spain) were germinated in 0.5-L pots with a mixture of peat and coconut fiber (2:1) substrate (Projar, Valencia, Spain). The plants were grown in a greenhouse under the following conditions: 16 h:8 h, 25°C:18°C light:dark photoperiod. Eight plants were challenged with TMGMV:PV-0124 (DSMZ, Braunschweig, Germany), and four were mock inoculated. Mechanical inoculation was carried out according to standard protocols. Nicotiana benthamiana leaves infected with TMGMV:PV-0124 and maintained frozen at −80°C were used as the inoculum source. The leaves were ground in a sterile mortar together with 30 mM phosphate buffer pH 8.0 (1 g in 5 mL). Carborundum was sprinkled on the expanded cotyledons of 7-day-old melon plants after germination, and the inoculum was applied by rubbing the cotyledons. Plants were checked periodically for symptoms for a period of 20 days. Cotyledons were collected 7 days postinoculation (dpi), and the second leaves were collected at 15 dpi, which were then placed in 1.5-mL tubes. RNA extraction and TMGMV detection in both cotyledons and second leaves were performed as mentioned in the previous sections.

### Cloning and sequencing of CABYV and ToLCNDV derived amplicons.

Complementary DNAs (cDNAs) to regions of the CABYV and ToLCNDV genomes were synthesized by RT-PCR. The primer pairs AB-882 (5′-CTCCTWCCGATATTGGCTCG-3′) and AB-883 (5′-AGCCCATTYHGCGCCRCAGT-3′) were designed based on the nucleotide sequence of isolate CABYV-N (NC003688) as reported previously (18), with modifications to amplify a fragment of 669 bp from ORF 2. The primers pairs AB-884 (5′-ATGTGGGATCCATTATTGCACGA-3′) and AB-885 (5′-TGCCTCGAGTAACATCACTAACA-3′) were designed on the nucleotide sequence of DNA-A of ToLCNDV (NC004611) to amplify the full AV2 gene. Primers were designed in conserved regions flanking variable genomic regions; even then, the conservation of the primer sequences was checked in the HTS data set.

RT and PCR were carried out using Expand Reverse Transcriptase (Roche, Mannheim, Germany) and NXT *Taq* PCR kit (EURx, Gdańsk, Poland), respectively, following the recommendations of the manufacturers. RT-PCR products were purified using the GENECLEAN kit (NZYTECH, Lisboa, Portugal). Purified fragments were cloned into a pGEM-T Easy vector (Promega, Madison, WI, USA) following the manufacturer’s protocol. Stellar competent cells (TaKaRa BIO INC, Shiga, Japan) were transformed and screened by colony PCR using M13 primers. The PCR products with the expected size were Sanger sequenced.

### Phylogenetic, population structure, and migration analyses.

Nucleic acid sequences were aligned using MUSCLE run in MEGA X (51) . Phylogenetic trees were constructed by using the minimum evolution method based on the K2P method for the AV2 gene of ToLCNDV and K2P with Gamma distribution method for the ORF2 of CABYV isolates. The Find best DNA/Protein models option was used to determine the best substitution model to construct the phylogenetic trees. The MEGA X software was used to calculate the nucleotide diversity (π) using K2P method for the AV2 gene and the K2P method with Gamma distribution for the ORF2. MEGA X was also used to calculate the frequency of dS and dN substitutions per site using the Pamilo-Bianchi-Li method. Codons under selection were determined using FUBAR through the use of the GTR substitution model by aligning sequences of the ORF2 of CABYV with its phylogenetic tree (54), and the algorithm was run in the Datamonkey web server (55). Genetic differentiation between populations was calculated using the Fixation index (Fst) in DnaSP6 v6 software (56). Fst is a measure of the average pairwise distances between pairs of individuals (haplotypes) in terms of allele frequencies. For between population comparisons, Fst consists of the difference between allele frequencies of two populations. The degree of differentiation (given by the Fst values) was classified as undifferentiated (Fst < 0.18), moderately differentiated (Fst = 0.18 to 0.30); or differentiated (Fst > 0.30). AMOVA and Mantel tests were carried out on Arlequin ver 3.5.2.2 (57). AMOVA estimates the genetic structure indices using information on the allelic content of haplotypes and their frequencies (58). The information on the differences in allelic content between haplotypes was entered as a matrix of Euclidean squared distances. The significance of the covariance components associated with the different possible levels of genetic structure (within populations, within groups of populations) was tested using nonparametric permutation procedures. The Mantel test computes correlation between two matrices (the population pairwise Fst matrix and the geographic matrix). Geographical distances were determined from the geographical coordinates of each sample, and were then converted to a matrix using Geographic distance matrix generator v1.2.3. Putative recombination sites were evaluated using the RDP4 package analysis for the CABYV isolates (59). The methods used to detect recombination events were: RDP, GENECONV, Maxi-mum χ2 (MaxChi), Chimera, BootScan, and SisterScan (SiScan) with default settings (59). To gain insight into the dissemination of CABYV and ToLCNDV across the sites and hosts, we reconstructed spatial transmission patterns using phylogenetic analysis in BEAST 1.10.4 (60). Four collection sites for CABYV (sites 1, 2, 3, and 4) and three for ToLCNDV (sites 1, 2, and 3) were coded as discrete states. Spatial diffusions between these sites (taking into consideration the host) were allowed to be asymmetric and model averaging was performed using Bayesian stochastic search variable selection (61). We used the same substitution model models as described above. Posterior distributions of parameters were estimated by Markov chain Monte Carlo sampling, with samples taken every 10,000 steps over 150,000,000 for CABYV data sets ad 130,000,000 for ToLCNDV data sets. Different combinations of clock models tree and tree priors (Clock model: strict versus relaxed; Tree prior: bayesian skyline, constant size and exponential growth) were used to perform BEAST analysis. After discarding the first 10% of the samples as burn-ins, the different parameters where checked by Tracer 1.7 (62) and the runs that showed sufficient sample size were retained. These runs showed similar migration patterns. The better supported pairwise diffusions were identified using Bayes factor in SPREAD 0.9.7.1 (63). Migration pathways were considered to be important when they yielded a Bayes factor greater than 3, and when the mean posterior value was greater than 0.5. Bayes factors were interpreted according to the guidelines of Kass and Raftery (64).

### Data availability.

Virus sequences were deposited in the GenBank database under the following accession numbers: CABYV (OM541599-OM541658) and ToLCNDV (OM475365-OM475424). Raw reads were submitted to the Sequence Read Archive (SRA) under the Bioproject ID no. PRJNA850108.
