# Supplementary material for: Genetic Differentiation and Migration Fluxes of Viruses from Melon Crops and Crop Edge Weeds
Source: J Virol. 2022 Aug 4;96(16):e00421-22. doi: 10.1128/jvi.00421-22 (PMC9400485; doi:10.1128/jvi.00421-22)
Supplement: Supplemental file 1 — Tables S1 to S9 and Fig. S1 to S4. Download jvi.00421-22-s0001.pdf, PDF file, 9.7 MB [file jvi.00421-22-s0001.pdf]

SUPPLEMENTARY TABLES AND FIGURES

TABLE S1. Summary of sequencing and reads' processing.

	**Melon**	***S. nigrum***	***M. sylvestris***	***E. elaterium (year 2020)***	***E. elaterium (year 2021)***
**Raw reads**	95,674,756	96,683,858	96,873,136	94,242,046	105,494,586
**Clean reads**	92,331,320	93,638,414	93,264,932	90,636,792	95,330,384
**Host mappings**	47,884,140	-	-	-	-
**Filter reads**	44,447,180	-	-	-	-
**Contigs**	31,876	68,239	64,738	47,717	16,106

TABLE S2 Primers and probes used for viral detection by qRT-PCR.

Virus	Target gene	Amplicon size	Primer/Probe	Sequence
**MNSV**	RdRp	101 bp	Forward	5'-CTCGCTGGGTTCTGTACTTC-3'
			Reverse	5'-CCTAAACAATACAGTTTGCGTGT-3'
			Probe	5'-FAM-CCCAGACAT/ZEN/GACCGAGTTTCCTCA-BHQ1-3'
**CMV**	CP	161 bp	Forward	5'-GAAGCTTGTTTCGCGCATTC-3'
			Reverse	5'-ACTGATAAACCAGTACCGGTG-3'
			Probe	5'-FAM-TGCCGAAATTTGATTCTACCGTGTGG-BHQ1-3'
**TMGMV**	CP	106 bp	Forward	GTAGCTATAAGGGCTTCAATCA
			Reverse	TGGTCCARACAAGTCCACTA
			Probe	5'6-FAM-TGAACTGGTTCGTGGAACTGGCAT-BHQ 3'
**CCYV**	RdRp	91 bp	Forward	5'-GGTTTACACACCCGGTGAGTTT-3'
			Reverse	5'-TGAAATTAGGGCTTGCTTCCA-3'
			Probe	5'-HEX-TCTACTCGGAAAAGGTGAAAGAGCTCGACC-BHQ1-3'
**ToLCNDV**	CP	144 bp	Forward	5'-CCAACCGGAACCCCTCAAGATT-3'
			Reverse	5'-TGCATACGTTCCTCCCGTCA-3'
			Probe	5'-HEX- TAGCACAGCCACGGTGAAGAACAT-BHQ-1-3'
**CmEV**	RdRp	129 bp	Forward	5'-TGGGAGTTCATCCAAACGTAATA-3'
			Reverse	5'-CCAGTCAACCGCATACCTTC-3'
			Probe	5'-TGGAAAGAAATGCATGAGGTGTGGAGA-3'
**WMV**	CP	102 bp	Forward	5'-ATTTCTCAGACGCAGCAGAA-3'
			Reverse	5'-CTAATTCCCTGTCTCTCAAATTTCT-3'
			Probe	5'-FAM-TGAGAAACTCTGAAAGTCCGTATATGCCT-IB® FQ-3'
**CABYV**	CP	58 bp	Forward	5'-GACGKTGGACCGCTAARCA-3'
			Reverse	5'-TGGTCTTCGGTTGCATCRTG-3'
			Probe	5'-FAM-ATCAACGGGATGGAATG-IB® FQ-3'

TABLE S3 Contigs of the virus species recovered from this studies with their % of nucleotide identity with the hit.

Contig ID	Length (nt)	Host[a]	Viral species	Fragments	% of nt[b]	Accession number[c]
k141_14574	5158	M	*Cucumis melo alphaendornavirus*	-	92.40 %	gi|1181747088|KX641269.1
k141_29085	8551		*Cucurbit chlorotic yellows virus*	RNA 1	99.88 %	gi|399142386|JN641883.1
k141_18754	8002			RNA 2	99.83 %	gi|289526328|AB523789.1
k141_14435	6154		*Watermelon mosaic virus*	-	98.25 %	gi|326468188|JF273461.1
k141_29706	3364		*Cucurbit aphid-borne yellows virus*	-	99.15 %	gi|2095906809|MW051363.1
k141_21575	1321		*Cucumber mosaic virus*	RNA 3	98.46 %	gi|25809284|AJ517802.1
k141_109	4199		*Melon necrotic spot virus*	-	97.14 %	gi|1695685772|MK604924.1
k141_10185	1948		*Tomato leaf curl new delhi virus*	DNA A	99.85 %	gi|1976373550|LC596380.1
k141_46034	643		*Tobacco mild green mosaic virus*	-	99.22 %	gi|1485815685|MH730970.1
k141_3324	2651	EE	*Cucurbit aphid-borne yellows virus*		97.39 %	gi|388325696|JF939814.1
k141_10185	1948		*Tomato leaf curl new delhi virus*	DNA A	99.85 %	gi|1976373550|LC596380.1
k141_47606	2405			DNA B	99.67 %	gi|1590128862|MH577635.1
k141_46034	643		*Tobacco mild green mosaic virus*	-	99.22 %	gi|1485815685|MH730970.1
k141_10942	2629		*Turnip yellows virus*	-	77.09 %	gi|428272515|JQ862472.1
k141_13206	9061		*Cucurbit yellow stunting disorder virus*	RNA 1	99.77 %	gi|146432248|EF547827.1
k141_1043	5043			RNA 2	99.84 %	gi|30691654|AY242078.1
k141_52574	3786	SN	*Tomato yellow mottle associated virus*		67.21 %	gi|2208169437|OM827247.1
k141_31540	7536		*Potato virus Y*		92.44 %	gi|1881044940|MT200665.1
k141_63224	767		*Moroccan pepper virus*		96.35 %	gi|392932145|JX197071.1
k141_61722	559		*Tomato yellow leaf curl Sardinia virus*		99.82 %	gi|531439372|KC953604.1
k141_24792	2082	MS	*Tomato yellow mottle associated virus*	-	65.03 %	gi|1158622146|KY075646.1
k141_3496	13122		*Eggpalnt mottled dwarf nucleorhabdovirus*	-	90.21 %	gi|594201519|KC905081.1
k141_3857	2579		*Potato virus Y*	-	93.52 %	gi|1881044940|MT200665.1
k141_49773	1500		*Malva vein clearing virus*	-	89.22 %	gi|1808995387|MN116683.1
k141_9830	1072		*Malva vein clearing virus*	-	83.44 %	gi|1808995387|MN116683.1
k141_37018	6933		*Malva vein clearing virus*	-	87.64 %	gi|1808995387|MN116683.1
k141_66058	2708		*Chickpea chlorotic dwarf virus*	-	85.57 %	gi|725826289|KM229817.1

[a]M; Melon, EE: *Ecballium elaterium*, SN: *Solanum nigrum*, MS: *Malva sylvestris.*

[b]Percentage of identity at nucleotide level of the contig with the viral species.

[c]The accession of the virus isolate that showed higher identity with the contig via website blastn.

TABLE S4 Description of the melon and weed samples' and collection sites.

Site ID	Localization	Sampling date	Species (Variety)	Sample ID	Pool ID	Symptoms
**First year survey**						
**Site 1**	**Cartagena**	17.06.2020	Melon (Charentais)	7	7Pool	yellowing
		17.06.2020		8		mosaic
		17.06.2020		9		yellowing
		24.06.2020		54	54Pool	yellowing
		24.06.2020		55		yellowing
		24.06.2020		56		Mosaic
		24.06.2020		57		yellowing
		24.06.2020		58		yellowing
		24.06.2020		59		yellowing
		24.06.2020		60		yellowing
		17.06.2020	*E. elaterium*	10	10Pool	Asymptomatic
		17.06.2020		11		Chlorosis in older leaves
		17.06.2020		12		Chlorosis in older leaves
		17.06.2020		13		Chlorosis in older leaves
		17.06.2020		14		Chlorosis in older leaves
		17.06.2020		15		Chlorosis in older leaves
		17.06.2020		16		Chlorosis in older leaves
		17.06.2020	*S. nigrum*	17	17Pool	Mosaic
		17.06.2020		18		Mosaic
		17.06.2020		19		Asymptomatic
		17.06.2020		20		chlorotic spots
		17.06.2020		21		Asymptomatic
		17.06.2020		22		Asymptomatic
		17.06.2020		23		Mosaic in young leaves
		17.06.2020	*M. sylvestris*	24	24Pool	Asymptomatic
		17.06.2020		25		Asymptomatic
		17.06.202		26		Leaf distortion
		17.06.2020		27		Asymptomatic
		17.06.2020		28		Asymptomatic
		17.06.2020		29		Yellowing
		17.06.2020		30		Asymptomatic
**Site 2**	**Cartagena**	17.06.2020	Melon (Charentais)	32	32Pool	Chlorotic spots
		07.07.2020		101	101Pool	mosaic
		07.07.2020		102		mosaic
		07.07.2020		103		mosaic
		07.07.2020		104		mosaic
		07.07.2020		105		nascent mosaic
		07.07.2020		106		yellowing
		07.07.2020		107		yellowing
		07.07.2020		108		yellowing
		07.07.2020		109		yellowing
		07.07.2020		110		New delhi symptoms
		17.06.2020	*E.elaterium*	33	33Pool	Yellowing
		17.06.2020		34		Yellowing
		17.06.2020		35		Yellowing

		17.06.2020		36		Yellowing
		17.06.2020		37		Yellowing
		17.06.2020		38		Yellowing
		17.06.2020		39		Asymptomatic
		17.06.2020	*S.nigrum*	40	40Pool	Asymptomatic
		17.06.2020		41		Asymptomatic
		17.06.2020		42		Mosaic
		17.06.2020		43		Chlorotic spots (insect)
		17.06.2020		44		Mosaic
		17.06.2020		45		Asymptomatic
		17.06.2020		46		Asymptomatic
		17.06.2020	*M. sylvestris*	47	47Pool	chlorotic spots (mites)
		17.06.2020		48		chlorotic spots (mites)
		17.06.2020		49		Asymptomatic
		17.06.2020		50		Asymptomatic
		17.06.2020		51		Asymptomatic
		17.06.2020		52		Asymptomatic
		17.06.2020		53		Asymptomatic
**Site 3**	**Torre-Pacheco**	24.06.2020	Melon (Piel de Sapo)	61	61Pool	mosaic
		24.06.2020		62		mosaic
		14.07.2020		143	143Pool	New delhi symptoms
		14.07.2020		144		mosaic
		14.07.2020		145		mosaic
		14.07.2020		146		mosaic
		14.07.2020		147		mosaic
		14.07.2020		148		yellowing
		14.07.2020		149		yellowing
		14.07.2020		150		
		14.07.2020		151		yellowing
		14.07.2020		152		yellowing
		24.06.2020	*E. elaterium*	63	63Pool	Yellowing
		24.06.2020		64		leaf malformation + yellowing
		24.06.2020		65		leaf malformation + yellowing
		24.06.2020		66		leaf malformation
		24.06.2020		67		leaf malformation
		24.06.2020		68		Asymptomatic
		24.06.2020		69		Asymptomatic
		24.06.2020	*S. nigrum*	70	70Pool	Nascent chlorosis
		24.06.2020		71		Asymptomatic
		24.06.2020		72		Asymptomatic
		24.06.2020		73		Nascent chlorosis
		24.06.2020		74		Asymptomatic
		24.06.2020		75		Asymptomatic
		24.06.2020		76		chlorotic spots
		24.06.2020	*M. sylvestris*	77	77Pool	Asymptomatic
		24.06.2020		78		Yellowing
		24.06.2020		79		Yellowing
		24.06.2020		80		Yellowing
		24.06.2020		81		Yellowing

Site 4	Lo Jurado	24.06.2020	Melon (Piel de Sapo)	82	82Pool	mosaic
		24.06.2020		83		mosaic
		24.06.2020		84		leaf curling,internods shortening, yellowing
		24.06.2020		85		leaf curling,internods shortening, yellowing
		24.06.2020		86		leaf curling,internods shortening, yellowing
		24.06.2020		87		yellowing + mosaic
		07.07.2020		111	111Pool	mosaic
		07.07.2020		112		mosaic
		07.07.2020		113		mosaic
		07.07.2020		114		yellowing
		07.07.2020		115		yellowing
		07.07.2020		116		yellowing
		07.07.2020		117		New delhi symptoms
		07.07.2020		118		New delhi symptoms
		07.07.2020		119		New delhi symptoms
		24.06.2020	*E. elaterium*	88	88Pool	Yellowing (insects)
		24.06.2020		89		Yellowing (insects)
		24.06.2020		90		Yellowing (insects)
		24.06.2020		91		Yellowing (insects)
		24.06.2020		92		Asymptomatic
		24.06.2020		93		Yellowing (insects)
		24.06.2020		94		Asymptomatic
		24.06.2020		95		Asymptomatic
		07.07.2020		120	120Pool	Asymptomatic
		07.07.2020		121		Asymptomatic
		07.07.2020		122		Asymptomatic
		07.07.2020		123		Asymptomatic
		07.07.2020		124		Asymptomatic
		07.07.2020		125		Asymptomatic
		07.07.2020		126		Asymptomatic
		07.07.2020		127		Chlorotic spots
Site 5	Aguillas	16.07.2020	Melon (Amarillo)	153	153Pool	mosaic
		16.07.2020		154		mosaic
		16.07.2020		155		mosaic
		16.07.2020		156		mosaic
		16.07.2020		157		mosaic
		16.07.2020		158		yellowing
		16.07.2020		159		yellowing
		16.07.2020		160		yellowing
		16.07.2020		161		yellowing
		16.07.2020		162		yellowing
		16.07.2020		163		yellowing
		16.07.2020		164		New delhi symptoms
Site 6	Santomera	16.07.2020	*E. elaterium*	172	172Pool	Asymptomatic
		16.07.2020		173		Yellowing
		16.07.2020		174		Asymptomatic
		16.07.2020		175		Asymptomatic
		16.07.2020		176		Asymptomatic
		16.07.2020		177		Asymptomatic

		16.07.2020		178		Asymptomatic
		16.07.2020		179		Asymptomatic
		16.07.2020		180		Yellowing
		16.07.2020		181		Chlorotic spots
		16.07.2020	*S. nigrum*	182	182Pool	Mosaic
		16.07.2020		183		Mosaic
		16.07.2020		184		Asymptomatic
		16.07.2020		185		Asymptomatic
		16.07.2020		186		Asymptomatic
		16.07.2020		187		Asymptomatic
		16.07.2020		188		Asymptomatic
		16.07.2020		189		Asymptomatic
		16.07.2020		190		Asymptomatic
		16.07.2020		191		Asymptomatic
		16.07.2020	*M. sylvestris*	192	192Pool	Yellowing
		16.07.2020		193		Asymptomatic
		16.07.2020		194		Asymptomatic
		16.07.2020		195		Asymptomatic
		16.07.2020		196		Asymptomatic
		16.07.2020		197		Asymptomatic
		16.07.2020		198		Yellowing
		16.07.2020		199		Asymptomatic
		16.07.2020		200		fungi symptoms
		16.07.2020		201		Asymptomatic
**Site 7**	**Villamanrique**	22.07.2020	Melon (Piel de Sapo)	202	202Pool	Mosaic (severity 4.5/5)
		22.07.2020		203		Mosaic (severity 3.5/5)
		22.07.2020		204		Mosaic (severity 2.5/5)
		22.07.2020		205		Mosaic (severity 2/5)
		22.07.2020		206		Mosaic (severity 2/5)
		22.07.2020		207		Mosaic (severity 2/5)
		22.07.2020		208		Mosaic (severity 1.5/5)
		22.07.2020		209		Yellowing
		22.07.2020		210		Yellowing
		22.07.2020		211		Yellowing
		22.05.2020	*E. elaterium*	1	1 Pool	Leaf malformation+ vein clearing
		22.05.2020		2		Vein clearing and malformation in the new leaves
		22.05.2020		3		Vein clearing in younger leaves
		22.05.2020		4		vein clearing and malformation of young leaves
		22.05.2020		5		Malformation of young leaves
		22.05.2020		6		chlorotic spots and malformation of new leaves
		22.07.2020		212	212Pool	Yellowing
		22.07.2020		213		Asymptomatic
		22.07.2020	*S. nigrum*	214	214Pool	Asymptomatic
		22.07.2020		215		Asymptomatic
		22.07.2020		216		Asymptomatic
		22.07.2020		217		Asymptomatic
		22.07.2020		218		Small chlorotic spots (mites probably)
		22.07.2020		219		Asymptomatic
		22.07.2020		220		Asymptomatic

**Site 8**	**Villaconejos**	22.07.2020	Melon (Piel de Sapo)	221	221Pool	Yellowing
		22.07.2020		222		Yellowing
		22.07.2020		223		Mosaic (severity 5/5)
		22.07.2020		224		Mosaic (severity 5/5)
		22.07.2020		225		Mosaic (severity 5/5)
		22.07.2020		226		Mosaic (severity 5/5)
		22.07.2020		227		Mosaic (severity 5/5)
		22.07.2020		228		Mosaic (severity 4/5)
		22.07.2020		229		Mosaic (severity 3/5)
		22.07.2020		230		Mosaic (severity 2/5)
		22.07.2020		231		Mosaic (severity 2/5)
		22.07.2020		232		Mosaic (severity 2/5)
		22.07.2020	*E. elaterium*	233	233Pool	Yellowing + leaf malformation
		22.07.2020		234		Asymptomatic
		22.07.2020		235		Asymptomatic
		22.07.2020		236		Asymptomatic
		22.07.2020		237		Asymptomatic
		22.07.2020		238		Asymptomatic
		22.07.2020		239		Asymptomatic
		22.07.2020	*M. sylvestris*	240	240Pool	Yellowing
		22.07.2020		241		Asymptomatic
		22.07.2020		242		Asymptomatic
		22.07.2020		243		Asymptomatic
		22.07.2020		244		Asymptomatic
		22.07.2020		245		Asymptomatic
		22.07.2020		246		Yellowing
**Second year survey**						
**Site 3**	**Torre-Pacheco**	26.07.2021	*E. elaterium*	255	255Pool	Asymtomatic
				256		Asymtomatic
				257		Asymtomatic
				258		Yellowing
				259		Asymtomatic
				260		Asymtomatic
				261		Asymtomatic
**Site 4**	**Lo-Jurado**	26.07.2021	*E. elaterium*	277	277Pool	Asymtomatic
				278		Asymtomatic
				279		Asymtomatic
				280		Asymtomatic
				281		Asymtomatic
				282		Asymtomatic
				283		Asymtomatic
				284		Yellowing
				285		Asymtomatic
				286		Asymtomatic
				287		Asymtomatic
				288		Asymtomatic
				289		Asymtomatic
				290		Asymtomatic
				291		Asymtomatic

				292		Asymtomatic
**Site 6**	**Santomera**	26.07.2021	*E. elaterium*	293	293Pool	Asymtomatic
				294		Asymtomatic
				295		Asymtomatic
				296		Asymtomatic
				297		Asymtomatic
				298		Asymtomatic
				299		Asymtomatic
				300		Asymtomatic
				301		Asymtomatic
				302		Asymtomatic

TABLE S5 Detection of TMGMV in Melon samples at 8 and 15 days post-inoculation (dpi) by qRT-PCR.

Plant ID	plant part	dpi	Ct value
**1**	Cotyledon	8	24.7
	Second Leaf	15	33.9
**2**	Cotyledon	8	28.7
	Second Leaf	15	-
**3**	Cotyledon	8	28.0
	Second Leaf	15	-
**4**	Cotyledon	8	25.5
	Second Leaf	15	-
**5**	Cotyledon	8	23.5
	Second Leaf	15	-
**6**	Cotyledon	8	25.0
	Second Leaf	15	-
**7**	Cotyledon	8	23.4
	Second Leaf	15	33.3
**8**	Cotyledon	8	24.4
	Second Leaf	15	30.3
**Negative control 1**	Cotyledon	8	-
	Second Leaf	15	-
**Negative control 2**	Cotyledon	8	-
	Second Leaf	15	-
**Negative control 3**	Cotyledon	8	-
	Second Leaf	15	-
**Negarive control 4**	Cotyledon	8	-
	Second Leaf	15	-

TABLE S6 Genetic diversity of Cucurbit aphid-borne yellows virus (CABYV) isolates in mean posterior non-synonymous (β) and synonymous (α) positions at each codon of the open reading frame (ORF) 2 for CABYV isolates from melon.

Site	α[a]	β[b]	B-α[c]	Prob[α>β][d]	Prob[α<β][e]	BayesFactor[α<β][f]
1	2.499	0.768	-1.732	0.675	0.266	0.609
2	8.692	0.344	-8.348	0.998	0.001	0.001
3	0.802	0.389	-0.413	0.608	0.330	0.829
4	1.313	0.358	-0.954	0.667	0.277	0.645
5	0.578	0.465	-0.113	0.527	0.408	1.159
6	0.595	0.410	-0.186	0.560	0.375	1.007
7	1.944	0.392	-1.552	0.821	0.142	0.279
8	2.699	0.381	-2.318	0.850	0.119	0.226
9	0.771	0.387	-0.385	0.605	0.333	0.838
10	0.867	0.388	-0.479	0.614	0.325	0.809
11	0.866	0.398	-0.468	0.608	0.331	0.831
12	0.741	0.412	-0.329	0.586	0.351	0.911
13	1.037	0.470	-0.567	0.730	0.220	0.476
14	0.874	0.471	-0.403	0.574	0.366	0.969
15	0.880	0.411	-0.469	0.603	0.337	0.854
16	4.007	0.358	-3.649	0.724	0.229	0.499
17	0.578	0.465	-0.113	0.527	0.408	1.159
18	0.829	0.889	0.060	0.360	0.580	2.325
19	0.661	0.423	-0.239	0.567	0.369	0.982
20	7.116	0.760	-6.356	0.919	0.055	0.098
21	0.682	0.405	-0.277	0.580	0.356	0.929
22	1.591	1.724	0.133	0.393	0.517	1.797
23	0.863	0.421	-0.442	0.595	0.344	0.883
24	6.082	0.396	-5.685	0.980	0.012	0.020
25	0.801	0.392	-0.409	0.606	0.332	0.837
26	0.596	0.391	-0.205	0.571	0.363	0.956
27	15.241	0.388	-14.853	0.999	0.001	0.001
28	2.865	4.139	1.274	0.254	0.616	2.693
29	0.595	0.403	-0.192	0.564	0.370	0.989
30	0.798	0.421	-0.377	0.589	0.350	0.904
31	1.749	0.475	-1.273	0.779	0.178	0.365
32	3.527	4.542	1.015	0.265	0.595	2.475
33	0.594	0.429	-0.165	0.548	0.386	1.058
34	2.628	0.381	-2.247	0.849	0.120	0.229
35	0.683	0.395	-0.288	0.587	0.349	0.901
36	0.863	0.421	-0.442	0.595	0.344	0.883
37	0.596	0.391	-0.205	0.571	0.363	0.956
38	0.627	0.392	-0.235	0.578	0.357	0.933
39	9.111	0.390	-8.721	0.993	0.004	0.006
40	11.847	0.384	-11.463	0.997	0.002	0.003
41	5.531	0.337	-5.195	0.964	0.026	0.045

42	5.129	0.376	-4.754	0.952	0.035	0.061
43	0.782	0.402	-0.381	0.598	0.340	0.868
44	4.225	0.386	-3.839	0.937	0.046	0.081
45	0.734	1.712	0.978	0.203	0.744	4.878
46	0.802	0.389	-0.413	0.608	0.330	0.829
47	3.423	0.378	-3.045	0.867	0.105	0.198
48	0.880	0.411	-0.469	0.603	0.337	0.854
49	0.778	0.454	-0.323	0.570	0.368	0.981
50	0.637	0.791	0.153	0.344	0.596	2.482
51	0.782	0.402	-0.381	0.598	0.340	0.868
52	6.367	0.399	-5.969	0.982	0.011	0.018
53	0.682	0.410	-0.271	0.577	0.359	0.942
54	0.780	0.420	-0.361	0.587	0.351	0.910
55	2.743	0.407	-2.337	0.840	0.127	0.244
56	0.769	0.406	-0.364	0.593	0.345	0.885
57	0.865	0.403	-0.462	0.605	0.334	0.844
58	0.769	0.406	-0.364	0.593	0.345	0.885
59	0.770	0.401	-0.369	0.596	0.342	0.873
60	0.783	0.397	-0.386	0.600	0.338	0.858
61	4.007	0.358	-3.649	0.724	0.229	0.499
62	0.866	0.395	-0.472	0.610	0.329	0.824
63	0.768	0.421	-0.347	0.584	0.354	0.920
64	1.500	0.376	-1.124	0.810	0.151	0.300
65	1.136	0.464	-0.671	0.741	0.211	0.448
66	2.013	0.406	-1.607	0.819	0.144	0.283
67	15.777	0.398	-15.378	0.998	0.001	0.002
68	0.738	1.017	0.280	0.330	0.611	2.644
69	1.434	0.454	-0.980	0.767	0.188	0.390
70	0.661	0.423	-0.239	0.567	0.369	0.982
71	0.769	0.408	-0.362	0.592	0.346	0.889
72	0.741	0.412	-0.329	0.586	0.351	0.911
73	0.595	0.423	-0.171	0.552	0.383	1.043
74	1.262	0.448	-0.814	0.758	0.196	0.411
75	2.955	0.365	-2.590	0.864	0.107	0.202
76	2.789	0.432	-2.356	0.833	0.133	0.258
77	0.683	0.389	-0.294	0.591	0.346	0.888
78	0.778	0.454	-0.323	0.570	0.368	0.981
79	10.178	0.361	-9.817	0.982	0.013	0.021
80	0.880	0.411	-0.469	0.603	0.337	0.854
81	0.769	0.406	-0.364	0.593	0.345	0.885
82	2.810	0.399	-2.411	0.846	0.122	0.233
83	7.091	0.337	-6.754	0.932	0.053	0.094
84	2.840	0.386	-2.454	0.852	0.117	0.222
85	1.316	1.083	-0.233	0.522	0.405	1.146
86	4.032	0.964	-3.068	0.484	0.464	1.453
87	0.725	0.405	-0.320	0.588	0.349	0.903
88	2.849	0.399	-2.450	0.848	0.121	0.231

89	0.595	0.423	-0.171	0.552	0.383	1.043
90	0.771	0.389	-0.382	0.603	0.334	0.844
91	0.772	0.385	-0.386	0.606	0.331	0.834
92	0.782	0.402	-0.381	0.598	0.340	0.868
93	0.771	0.389	-0.382	0.603	0.334	0.844
94	11.063	0.386	-10.677	0.999	0.000	0.001
95	2.131	0.365	-1.766	0.841	0.125	0.241
96	0.767	0.427	-0.340	0.581	0.357	0.934
97	4.005	0.421	-3.585	0.923	0.056	0.100
98	11.257	0.346	-10.911	0.998	0.001	0.002
99	0.802	0.387	-0.416	0.609	0.329	0.824
100	10.808	0.371	-10.437	0.996	0.002	0.004
101	0.878	0.431	-0.446	0.592	0.348	0.896
102	0.778	0.454	-0.323	0.570	0.368	0.981
103	11.352	0.381	-10.971	0.997	0.002	0.003
104	2.898	0.386	-2.513	0.854	0.115	0.219
105	0.878	0.431	-0.446	0.592	0.348	0.896
106	8.952	0.346	-8.605	0.982	0.013	0.022
107	3.257	1.058	-2.198	0.397	0.548	2.035
108	0.874	0.471	-0.403	0.574	0.366	0.969
109	4.475	0.351	-4.125	0.949	0.037	0.065
110	0.770	0.401	-0.369	0.596	0.342	0.873
111	2.729	0.365	-2.364	0.858	0.112	0.213
112	10.441	0.363	-10.078	0.996	0.002	0.004
113	7.126	0.407	-6.719	0.966	0.024	0.041
114	0.575	1.785	1.210	0.170	0.783	6.056
115	0.798	0.427	-0.371	0.585	0.353	0.917
116	1.034	0.471	-0.563	0.730	0.221	0.476
117	0.802	0.387	-0.416	0.609	0.329	0.824
118	0.596	0.391	-0.205	0.571	0.363	0.956
119	0.801	0.392	-0.409	0.606	0.332	0.837
120	2.321	3.931	1.609	0.232	0.654	3.180
121	0.578	0.465	-0.113	0.527	0.408	1.159
122	5.088	0.349	-4.738	0.956	0.032	0.055
123	0.771	0.387	-0.385	0.605	0.333	0.838
124	0.878	0.431	-0.446	0.592	0.348	0.896
125	4.042	0.440	-3.602	0.685	0.268	0.615
126	0.629	0.495	-0.134	0.530	0.407	1.152
127	0.731	0.791	0.060	0.363	0.577	2.297
128	13.783	0.369	-13.414	0.998	0.001	0.002
129	3.701	0.682	-3.019	0.530	0.418	1.209
130	1.313	0.358	-0.954	0.667	0.277	0.645
131	0.662	0.409	-0.253	0.575	0.361	0.949
132	0.596	0.389	-0.207	0.573	0.361	0.950
133	0.863	0.421	-0.442	0.595	0.344	0.883
134	0.866	0.399	-0.467	0.607	0.332	0.834
135	7.042	0.340	-6.702	0.974	0.019	0.032

136	11.396	0.386	-11.010	0.999	0.000	0.001
137	9.553	0.333	-9.220	0.996	0.002	0.004
138	0.863	0.421	-0.442	0.595	0.344	0.883
139	4.042	0.440	-3.602	0.685	0.268	0.615
140	0.866	0.395	-0.472	0.610	0.329	0.824
141	0.595	0.423	-0.171	0.552	0.383	1.043
142	0.801	0.392	-0.409	0.606	0.332	0.837
143	0.863	0.421	-0.442	0.595	0.344	0.883
144	4.042	0.440	-3.602	0.685	0.268	0.615
145	0.770	0.401	-0.369	0.596	0.342	0.873
146	0.595	0.403	-0.192	0.564	0.370	0.989
147	0.739	0.867	0.127	0.351	0.589	2.414
148	5.826	0.365	-5.461	0.961	0.028	0.049
149	0.682	0.410	-0.271	0.577	0.359	0.942
150	4.158	0.676	-3.482	0.541	0.408	1.158
151	34.881	0.473	-34.408	1.000	0.000	0.000
152	0.768	0.421	-0.347	0.584	0.354	0.920
153	0.629	0.495	-0.134	0.530	0.407	1.152
154	16.165	1.398	-14.767	0.972	0.008	0.013
155	0.769	0.406	-0.364	0.593	0.345	0.885
156	1.144	1.126	-0.018	0.493	0.433	1.284
157	0.802	0.387	-0.416	0.609	0.329	0.824
158	2.567	0.381	-2.185	0.847	0.121	0.232
159	0.682	0.410	-0.271	0.577	0.359	0.942
160	1.268	0.450	-0.819	0.758	0.196	0.410
161	0.817	0.764	-0.053	0.381	0.559	2.128
162	11.543	4.531	-7.012	0.650	0.161	0.323
163	0.732	0.732	-0.000	0.377	0.562	2.159
164	0.802	0.387	-0.416	0.609	0.329	0.824
165	0.769	0.406	-0.364	0.593	0.345	0.885
166	7.121	0.347	-6.774	0.991	0.006	0.010
167	1.242	0.453	-0.789	0.755	0.198	0.416
168	5.616	0.765	-4.850	0.890	0.076	0.139
169	0.662	0.409	-0.253	0.575	0.361	0.949
170	3.044	0.385	-2.659	0.858	0.112	0.212
171	4.042	0.440	-3.602	0.685	0.268	0.615
172	2.859	0.430	-2.430	0.894	0.079	0.144
173	0.648	1.352	0.704	0.213	0.733	4.611
174	16.203	0.380	-15.823	0.998	0.001	0.001
175	5.539	0.341	-5.198	0.913	0.068	0.123
176	2.645	0.373	-2.272	0.853	0.116	0.220
177	3.567	0.372	-3.194	0.873	0.100	0.187
178	0.866	0.395	-0.472	0.610	0.329	0.824
179	0.768	0.421	-0.347	0.584	0.354	0.920
180	0.770	0.401	-0.369	0.596	0.342	0.873
181	0.866	0.399	-0.467	0.607	0.332	0.834
182	0.579	0.777	0.198	0.330	0.610	2.630

183	7.896	0.334	-7.561	0.994	0.004	0.006
184	3.436	0.395	-3.040	0.861	0.110	0.207
185	0.661	0.438	-0.223	0.559	0.377	1.017
186	1.974	0.373	-1.602	0.832	0.133	0.257
187	1.274	0.450	-0.824	0.758	0.196	0.409
188	3.170	0.425	-2.745	0.904	0.071	0.129
189	0.800	0.407	-0.392	0.596	0.342	0.873
190	0.874	0.471	-0.403	0.574	0.366	0.969
191	0.725	0.405	-0.320	0.588	0.349	0.903
192	0.768	0.421	-0.347	0.584	0.354	0.920
193	2.095	0.374	-1.722	0.836	0.130	0.250
194	0.767	0.427	-0.340	0.581	0.357	0.934
195	4.337	0.391	-3.946	0.888	0.088	0.162
196	3.506	0.446	-3.060	0.847	0.122	0.233
197	3.445	0.387	-3.058	0.866	0.106	0.199
198	0.683	0.387	-0.297	0.592	0.344	0.880
199	0.767	0.427	-0.340	0.581	0.357	0.934
200	0.866	0.395	-0.472	0.610	0.329	0.824
201	17.601	0.391	-17.210	0.998	0.001	0.001
202	0.683	0.389	-0.294	0.591	0.346	0.888
203	0.802	0.387	-0.416	0.609	0.329	0.824
204	0.638	0.751	0.113	0.353	0.586	2.384
205	0.801	0.392	-0.409	0.606	0.332	0.837
206	2.934	0.802	-2.132	0.687	0.255	0.576

[a]Mean posterior synonymous substitution rate at a site.

[b]Mean posterior non-synonymous substitution rate at a site.

[c]Mean posterior beta-alpha.

[d]Posterior probability of negative selection at a site.

[e]Posterior probability of positive selection at a site.

[f]Empirical Bayes Factor for positive selection at a site.

TABLE S7 Genetic diversity of Cucurbit aphid-borne yellows virus (CABYV) isolates in mean posterior non-synonymous (β) and synonymous (α) positions at each codon of the open reading frame (ORF) 2 for CABYV isolates from *E. elaterium*.

Site	α	β	Β-α	Prob[α>β]	Prob[α<β]	BayesFactor[α<β]
1	0.648	0.332	-0.316	0.619	0.315	0.860
2	6.415	0.218	-6.197	0.998	0.001	0.002
3	0.694	0.315	-0.379	0.640	0.295	0.782
4	1.271	0.274	-0.997	0.723	0.222	0.534
5	0.491	0.408	-0.083	0.523	0.407	1.285
6	0.542	0.337	-0.205	0.589	0.341	0.969
7	0.542	0.336	-0.206	0.590	0.340	0.965
8	4.215	0.255	-3.960	0.962	0.028	0.053
9	1.804	0.285	-1.519	0.868	0.103	0.215
10	0.717	0.321	-0.396	0.638	0.297	0.792
11	0.717	0.321	-0.396	0.638	0.297	0.791
12	0.646	0.339	-0.307	0.614	0.320	0.881
13	0.440	0.407	-0.033	0.496	0.431	1.418
14	0.725	0.398	-0.327	0.590	0.347	0.993
15	0.738	0.334	-0.404	0.632	0.304	0.818
16	3.510	0.559	-2.951	0.585	0.365	1.076
17	0.491	0.408	-0.083	0.523	0.407	1.285
18	0.577	6.090	5.513	0.007	0.980	93.727
19	1.412	0.333	-1.079	0.825	0.139	0.302
20	0.638	0.328	-0.309	0.620	0.314	0.854
21	0.607	0.336	-0.271	0.608	0.325	0.900
22	0.594	3.686	3.091	0.062	0.909	18.610
23	0.641	0.682	0.041	0.377	0.563	2.406
24	0.479	0.394	-0.084	0.524	0.404	1.270
25	7.184	0.220	-6.963	0.996	0.002	0.004
26	0.544	0.319	-0.225	0.604	0.326	0.904
27	6.895	0.228	-6.667	0.996	0.003	0.005
28	25.754	0.379	-25.375	1.000	0.000	0.000
29	0.543	0.334	-0.208	0.591	0.339	0.959
30	0.687	0.351	-0.335	0.613	0.323	0.892
31	2.534	0.834	-1.700	0.755	0.186	0.429
32	0.734	0.354	-0.379	0.618	0.319	0.876
33	0.541	0.357	-0.184	0.574	0.357	1.037
34	0.648	0.332	-0.316	0.619	0.315	0.860
35	0.608	0.329	-0.279	0.613	0.320	0.879
36	0.711	0.347	-0.364	0.619	0.317	0.869
37	1.235	0.302	-0.932	0.835	0.129	0.278
38	3.176	0.262	-2.914	0.947	0.039	0.076
39	1.260	0.397	-0.863	0.782	0.177	0.401
40	3.144	0.289	-2.855	0.938	0.046	0.090
41	5.531	0.569	-4.962	0.921	0.058	0.115

42	0.541	0.355	-0.186	0.575	0.355	1.030
43	2.012	0.294	-1.718	0.868	0.103	0.214
44	7.763	0.242	-7.521	0.999	0.001	0.001
45	0.680	0.343	-0.337	0.617	0.318	0.871
46	0.628	0.618	-0.010	0.400	0.539	2.190
47	5.023	0.245	-4.778	0.972	0.020	0.039
48	2.328	0.298	-2.030	0.874	0.098	0.204
49	0.673	0.381	-0.292	0.591	0.345	0.984
50	1.220	0.639	-0.582	0.625	0.311	0.845
51	0.683	0.324	-0.359	0.631	0.304	0.815
52	6.180	0.264	-5.916	0.995	0.003	0.005
53	0.606	0.342	-0.264	0.603	0.330	0.921
54	0.680	0.343	-0.337	0.617	0.318	0.871
55	4.149	0.274	-3.875	0.957	0.032	0.062
56	0.647	0.333	-0.314	0.618	0.316	0.865
57	0.714	0.333	-0.381	0.629	0.307	0.828
58	0.647	0.333	-0.314	0.618	0.316	0.865
59	0.648	0.332	-0.316	0.619	0.315	0.860
60	0.662	0.324	-0.338	0.628	0.307	0.827
61	4.017	0.267	-3.750	0.789	0.169	0.380
62	10.227	0.234	-9.993	1.000	0.000	0.000
63	0.644	0.353	-0.292	0.604	0.331	0.924
64	0.517	0.615	0.098	0.362	0.577	2.553
65	0.440	0.407	-0.033	0.496	0.431	1.418
66	19.401	1.142	-18.259	0.998	0.000	0.000
67	6.486	0.234	-6.252	0.994	0.004	0.008
68	0.673	0.381	-0.292	0.591	0.345	0.984
69	0.491	0.408	-0.083	0.523	0.407	1.285
70	1.412	0.333	-1.079	0.825	0.139	0.302
71	0.647	0.335	-0.313	0.617	0.317	0.869
72	0.599	0.667	0.068	0.372	0.568	2.455
73	5.278	0.247	-5.031	0.987	0.008	0.016
74	7.869	0.258	-7.611	1.000	0.000	0.000
75	0.717	0.321	-0.396	0.638	0.297	0.792
76	0.673	0.381	-0.292	0.591	0.345	0.984
77	0.566	0.615	0.049	0.381	0.558	2.357
78	0.673	0.381	-0.292	0.591	0.345	0.984
79	11.983	0.252	-11.731	1.000	0.000	0.000
80	9.667	0.587	-9.080	0.988	0.007	0.013
81	0.647	0.333	-0.314	0.618	0.316	0.865
82	15.713	0.306	-15.407	1.000	0.000	0.000
83	18.284	0.300	-17.984	1.000	0.000	0.000
84	2.028	0.302	-1.725	0.864	0.106	0.222
85	2.040	0.361	-1.679	0.886	0.087	0.177
86	4.066	0.364	-3.702	0.732	0.225	0.542
87	0.638	0.328	-0.309	0.620	0.314	0.854
88	0.687	0.351	-0.335	0.613	0.323	0.892

89	0.541	0.355	-0.186	0.575	0.355	1.030
90	0.650	0.316	-0.334	0.631	0.302	0.810
91	0.651	0.315	-0.336	0.633	0.301	0.806
92	5.084	0.242	-4.842	0.974	0.019	0.036
93	0.650	0.316	-0.334	0.631	0.302	0.810
94	7.164	0.236	-6.928	0.998	0.001	0.002
95	1.337	0.604	-0.733	0.655	0.285	0.744
96	0.644	0.354	-0.290	0.602	0.332	0.930
97	0.491	0.408	-0.083	0.523	0.407	1.285
98	7.713	0.206	-7.507	1.000	0.000	0.000
99	2.210	0.277	-1.933	0.883	0.091	0.186
100	4.761	0.255	-4.506	0.970	0.022	0.042
101	0.657	0.715	0.059	0.372	0.569	2.466
102	1.949	0.348	-1.601	0.838	0.129	0.277
103	0.578	0.354	-0.224	0.588	0.344	0.983
104	2.031	0.302	-1.728	0.865	0.106	0.222
105	0.734	0.354	-0.379	0.618	0.319	0.876
106	1.821	0.608	-1.213	0.692	0.253	0.632
107	2.010	0.803	-1.207	0.416	0.529	2.099
108	0.725	0.398	-0.327	0.590	0.347	0.993
109	3.392	0.256	-3.136	0.952	0.035	0.068
110	0.648	0.332	-0.316	0.619	0.315	0.860
111	4.271	0.240	-4.031	0.966	0.025	0.047
112	1.222	0.320	-0.901	0.822	0.140	0.306
113	2.205	0.343	-1.861	0.849	0.120	0.255
114	0.541	0.355	-0.186	0.575	0.355	1.030
115	0.687	0.353	-0.333	0.611	0.324	0.898
116	17.426	0.390	-17.036	1.000	0.000	0.000
117	2.057	0.280	-1.777	0.878	0.095	0.196
118	5.641	0.216	-5.425	0.992	0.005	0.010
119	0.693	0.320	-0.373	0.636	0.299	0.798
120	0.680	0.343	-0.337	0.617	0.318	0.871
121	0.984	0.388	-0.596	0.761	0.194	0.450
122	1.260	0.920	-0.340	0.486	0.438	1.457
123	0.597	0.607	0.010	0.395	0.544	2.228
124	0.665	0.701	0.036	0.378	0.562	2.403
125	4.066	0.364	-3.702	0.732	0.225	0.542
126	1.147	0.855	-0.292	0.547	0.385	1.169
127	1.242	2.643	1.401	0.165	0.742	5.392
128	2.239	0.293	-1.945	0.874	0.098	0.204
129	4.017	0.267	-3.750	0.789	0.169	0.380
130	0.967	0.783	-0.183	0.361	0.581	2.589
131	0.580	0.336	-0.244	0.601	0.331	0.924
132	0.544	0.313	-0.232	0.608	0.321	0.884
133	0.711	0.347	-0.364	0.619	0.317	0.869
134	2.192	0.296	-1.896	0.872	0.100	0.208
135	0.651	0.315	-0.336	0.633	0.301	0.806

136	4.385	0.271	-4.115	0.961	0.029	0.056
137	7.934	0.214	-7.720	0.999	0.000	0.001
138	0.711	0.347	-0.364	0.619	0.317	0.869
139	4.066	0.364	-3.702	0.732	0.225	0.542
140	5.169	0.237	-4.932	0.974	0.019	0.035
141	1.217	0.338	-0.879	0.811	0.151	0.331
142	0.693	0.320	-0.373	0.636	0.299	0.798
143	5.540	0.257	-5.283	0.974	0.019	0.036
144	4.066	0.364	-3.702	0.732	0.225	0.542
145	6.420	0.226	-6.194	0.994	0.004	0.007
146	2.790	0.279	-2.511	0.936	0.047	0.093
147	0.680	0.343	-0.337	0.617	0.318	0.871
148	0.606	0.342	-0.264	0.603	0.330	0.921
149	0.563	0.667	0.104	0.359	0.580	2.586
150	4.017	0.267	-3.750	0.789	0.169	0.380
151	5.309	0.263	-5.046	0.972	0.021	0.039
152	0.644	0.353	-0.292	0.604	0.331	0.924
153	5.154	0.287	-4.868	0.982	0.012	0.022
154	0.717	0.321	-0.396	0.638	0.297	0.791
155	0.647	0.333	-0.314	0.618	0.316	0.865
156	1.758	0.366	-1.392	0.873	0.097	0.200
157	0.696	0.309	-0.386	0.645	0.290	0.765
158	5.057	0.237	-4.820	0.987	0.009	0.016
159	1.574	0.318	-1.256	0.840	0.126	0.271
160	7.327	0.250	-7.078	0.999	0.000	0.001
161	5.112	0.239	-4.873	0.974	0.019	0.037
162	1.982	0.312	-1.670	0.858	0.112	0.236
163	4.562	0.233	-4.329	0.971	0.021	0.040
164	1.973	0.281	-1.692	0.875	0.097	0.202
165	7.428	0.585	-6.842	0.978	0.013	0.025
166	6.545	0.570	-5.976	0.974	0.016	0.030
167	6.343	0.253	-6.090	0.998	0.001	0.001
168	10.065	0.250	-9.815	1.000	0.000	0.000
169	1.498	0.314	-1.184	0.840	0.126	0.270
170	15.643	0.302	-15.342	1.000	0.000	0.000
171	4.066	0.364	-3.702	0.732	0.225	0.542
172	1.668	0.786	-0.881	0.696	0.239	0.587
173	1.270	2.911	1.641	0.152	0.756	5.805
174	9.017	0.237	-8.781	0.998	0.001	0.002
175	7.870	0.211	-7.659	0.999	0.000	0.001
176	9.446	0.234	-9.212	1.000	0.000	0.000
177	5.040	0.240	-4.800	0.973	0.020	0.037
178	0.717	0.318	-0.399	0.640	0.295	0.784
179	0.644	0.353	-0.292	0.604	0.331	0.924
180	0.648	0.332	-0.316	0.619	0.315	0.860
181	2.301	0.294	-2.007	0.875	0.098	0.202
182	1.243	0.317	-0.926	0.825	0.138	0.299

183	6.514	0.214	-6.300	0.995	0.003	0.006
184	0.530	3.266	2.736	0.041	0.933	26.168
185	0.578	0.361	-0.217	0.583	0.349	1.004
186	1.423	0.293	-1.131	0.850	0.117	0.248
187	1.830	0.354	-1.477	0.881	0.090	0.185
188	5.199	0.270	-4.929	0.991	0.005	0.010
189	2.067	0.302	-1.765	0.866	0.105	0.220
190	0.725	0.398	-0.327	0.590	0.347	0.993
191	1.920	0.299	-1.621	0.863	0.107	0.224
192	0.597	0.695	0.098	0.362	0.578	2.564
193	6.897	0.547	-6.350	0.978	0.014	0.027
194	0.649	0.356	-0.293	0.602	0.332	0.931
195	5.051	0.264	-4.787	0.969	0.022	0.043
196	5.285	0.299	-4.986	0.964	0.026	0.049
197	2.363	0.298	-2.065	0.875	0.097	0.202
198	0.614	0.316	-0.298	0.624	0.308	0.834
199	0.649	0.356	-0.293	0.602	0.332	0.931
200	0.724	0.320	-0.404	0.640	0.296	0.786
201	5.154	0.251	-4.903	0.972	0.021	0.040
202	1.628	0.295	-1.333	0.856	0.113	0.238
203	2.047	0.282	-1.766	0.876	0.096	0.199
204	1.435	0.304	-1.130	0.843	0.123	0.263
205	5.102	0.238	-4.864	0.975	0.018	0.034
206	5.664	0.549	-5.115	0.966	0.022	0.042

[a]Mean posterior synonymous substitution rate at a site.

[b]Mean posterior non-synonymous substitution rate at a site.

[c]Mean posterior beta-alpha.

[d]Posterior probability of negative selection at a site.

[e]Posterior probability of positive selection at a site.

[f]Empirical Bayes Factor for positive selection at a site.

TABLE S8 Statistically supported migration rates of Cucurbit aphid-borne yellows virus estimated from the ORF2 among sites 1, 2, 3 and 4.

From (host[a])	To (host[a])	Mean migration rate[b]	Indicator[c]	Bayes Factor[d]
Site2 (EE)	Site3 (EE)	1.07	0.97	158.17
Site1 (M)	Site3 (M)	0.98	0.91	41.95
Site2 (M)	Site3 (M)	1.83	0.87	28.89
Site2 (EE)	Site4 (EE)	0.99	0.87	28.04
Site1 (EE)	Site4 (EE)	1.07	0.73	11.28
Site1 (M)	Site4 (M)	1.22	0.57	5.4
Site3 (M)	Site4 (M)	0.99	0.56	5.31

[a]EE: *Ecballium elaterium*; M: Melon.

[b]Unit: migration event per lineage per year.

[c]Posterior probability of observing a non-zero migration rate in the sampled trees.

[d]Only statistically supported migrations with indicator values > 0,50 and BF > 3 are shown.

TABLE S9 Statistically supported migration rates of Tomato leaf curl New Delhi virus estimated from the AV2 gene among sites 2, 3, and 4.

From (host)	To (host)	Mean migration rate	Indicator	Bayes Factor
Site2 (EE)	Site4 (EE)	1.01	0,91	30,24
Site4 (M)	Site4 (EE)	1.34	0,83	15,17
Site3 (EE)	Site4 (EE)	1.01	0,53	3,61
Site3 (M)	Site4 (EE)	1.02	0,51	3,26
Site2 (M)	Site4 (EE)	0.97	0,51	3,23

[a]EE:*Ecballium elaterium*, M: Melon.

[b]Unit: migration event per lineage per year.

[c]Posterior probability of observing a non-zero migration rate in the sampled trees.

[d]Only statistically supported migrations with indicator values > 0,50 and BF > 3 are shown.

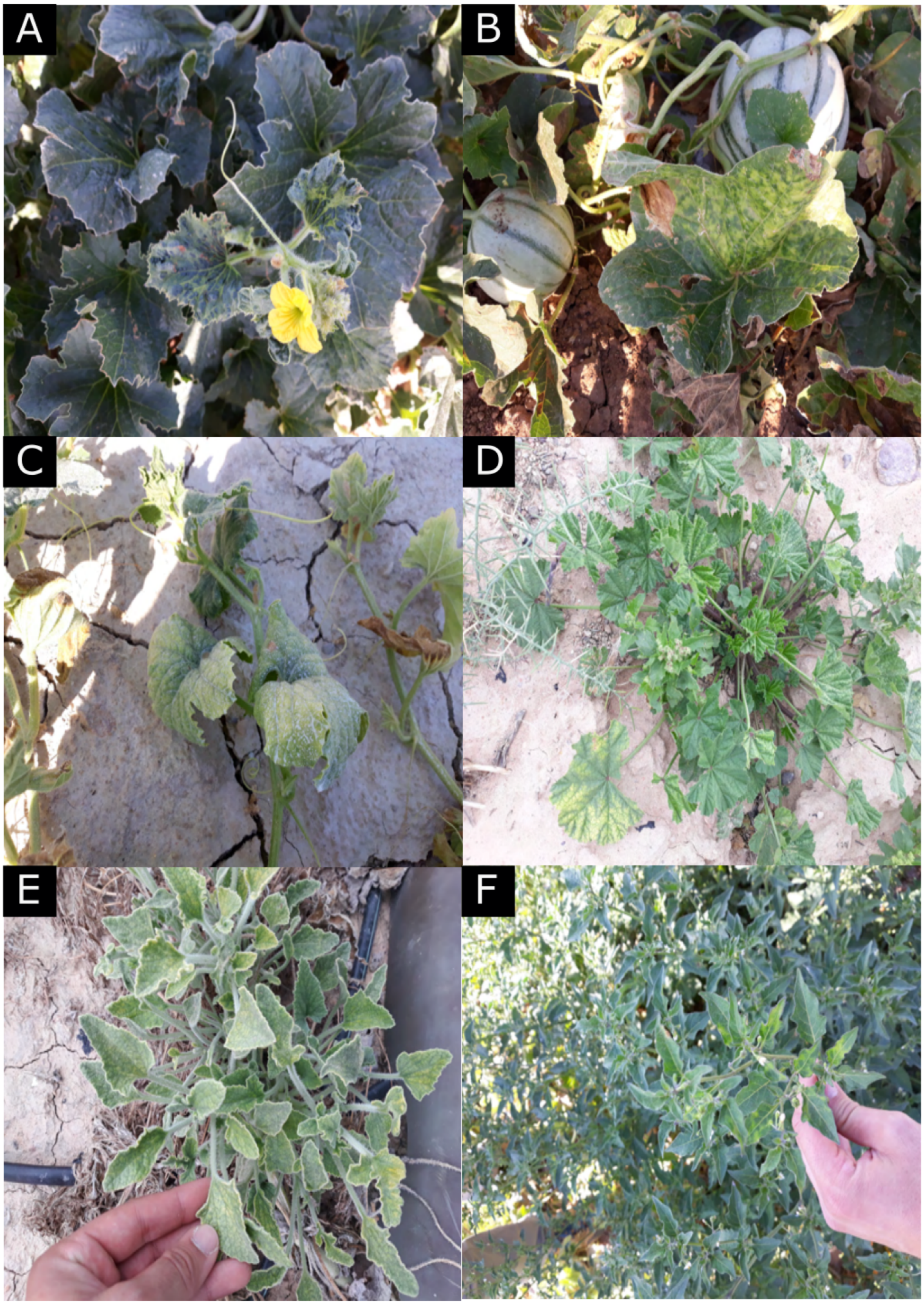

FIG S1 Symptoms in melon and associated weed plants collected during surveys. In melon, mosaics in the upper leaves **(A)** and yellowing on the basal leaves of older plants **(B)** were more frequent, whereas leaf-curling, leaf area reduction and internode shortening, and severe stunting **(C)** were less frequent. Weeds were almost asymptomatic, with few exceptions where we noticed general chlorosis in *Malva sylvestris* **(D)** and *Ecballium elaterium* **(E)** and mosaics in the upper leaves of *Solanum nigrum* **(F)**.

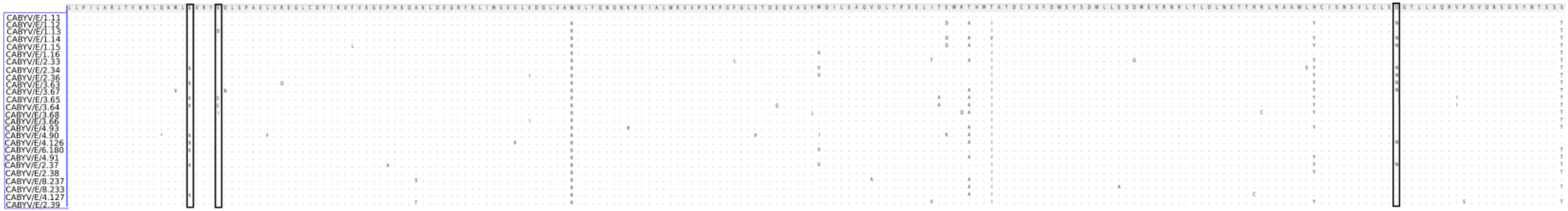

FIG S2 Alignment of CABYV amino acid sequences from *E. elaterium* isolates showing the codons under positive selection in ORF2. A rectangular box represents the amino acid putatively under positive selection.

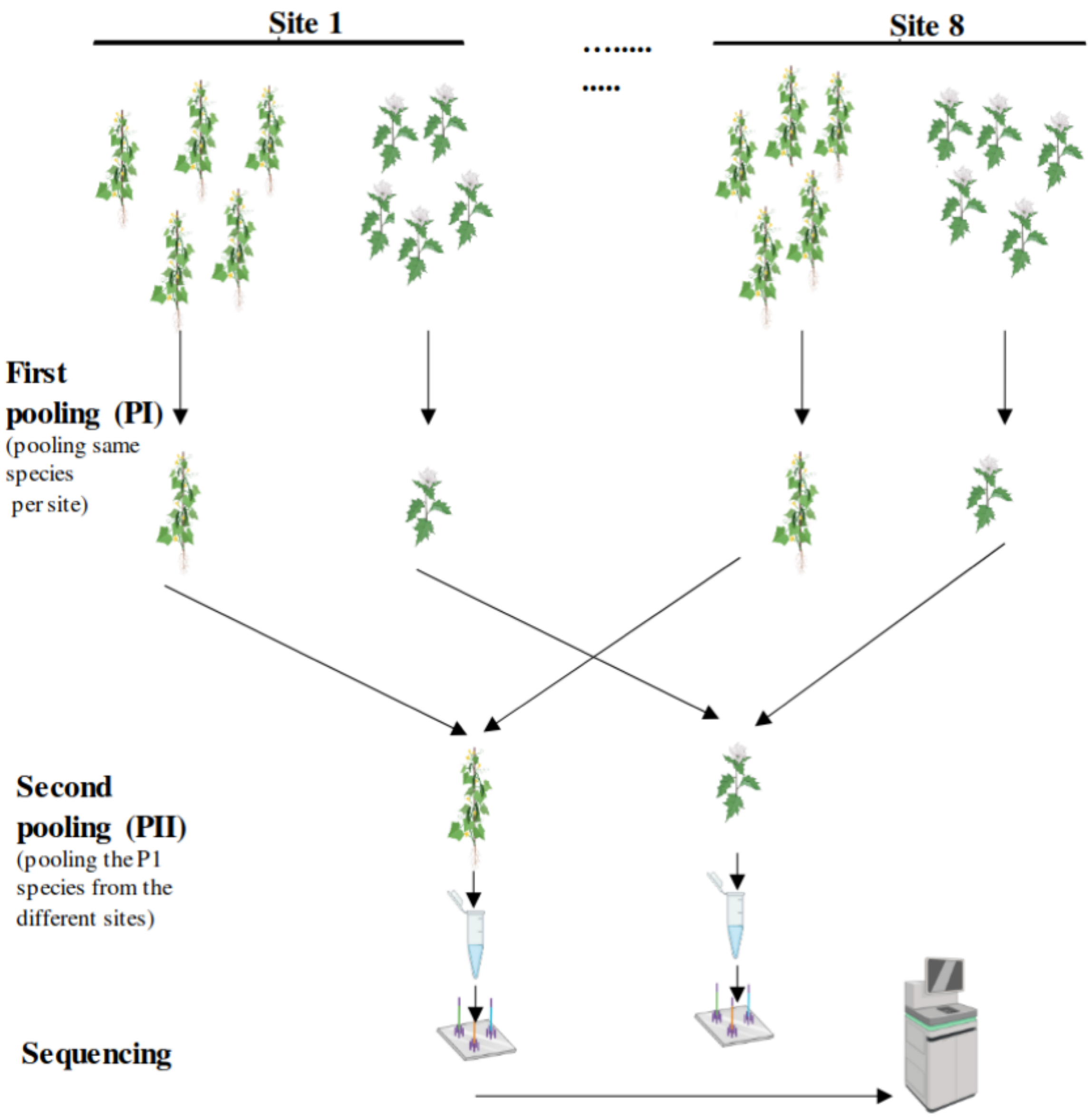

FIG S3 Schematic representation showing the pooling and sequencing strategy. Samples were pooled in a first stage by species and site. A second pool was constructed by pooling the first pools by species.
